# HDAC6 and USP9X Control Glutamine Metabolism by Stabilizing GS to Promote Glioblastoma Tumorigenesis

**DOI:** 10.1002/advs.202501553

**Published:** 2025-03-31

**Authors:** Go Woon Kim, Minhae Cha, Hien Thi My Ong, Jung Yoo, Yu Hyun Jeon, Sang Wu Lee, Soo Yeon Oh, Min‐Jung Kang, Youngsoo Kim, So Hee Kwon

**Affiliations:** ^1^ College of Pharmacy Yonsei Institute of Pharmaceutical Sciences Yonsei University Incheon 21983 Republic of Korea; ^2^ Center for Advanced Biomolecular Recognition Korea Institute of Science and Technology Seoul 02792 Republic of Korea; ^3^ Division of Bio‐Medical Science & Technology KIST School University of Science and Technology Seoul 02792 Republic of Korea

**Keywords:** glioblastoma, glutamine metabolism, GS, HDAC6, USP9X

## Abstract

Glioblastoma (GBM) is the most common and the deadliest brain cancer. Glutamine anabolism mediated by glutamine synthetase (GS) is beneficial for GBM cell growth, especially under glutamine deprivation. However, the molecular mechanism underlying GS homeostasis in GBM remains undisclosed. Here, it is reported that histone deacetylase 6 (HDAC6) promotes GS deacetylation, stabilizing it via ubiquitin‐mediated pathway. It is found that deubiquitination of GS is modulated by ubiquitin‐specific peptidase 9, X‐linked (USP9X). USP9X stabilizes GS by removing its K48‐linked polyubiquitination on lysine 91 and 103. Accordingly, targeting HDAC6 and USP9X in vitro and in vivo represses GBM tumorigenesis by decreasing GS stability. Metabolic analysis shows that silencing HDAC6 and USP9X disrupts de novo nucleotide synthesis, thereby attenuating GBM cell growth. Furthermore, GS modulation by targeting HDAC6 and USP9X restrains the self‐renewal capacity. These results suggest that HDAC6 and USP9X are crucial epigenetic enzymes that promote GBM tumorigenesis by modulating glutamine metabolism.

## Graphical abstract

Glioblastoma (GBM) growth relies on glutamine synthetase (GS), which is stabilized by histone deacetylase 6 (HDAC6) and deubiquitinated by ubiquitin‐specific peptidase 9, X‐linked (USP9X). HDAC6 promotes GS deacetylation, while USP9X removes its K48‐linked polyubiquitination, enhancing GS stability. Targeting HDAC6 and USP9X suppresses GBM growth, disrupts nucleotide synthesis, and impairs GBM self‐renewal, highlighting their critical role in GBM tumorigenesis.

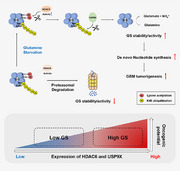

## 1 Introduction

Glioblastoma (GBM), the most aggressive and common malignant brain tumor that starts in astrocytes, displays extensive metabolic reprogramming.^[^ ^1^, ^2^ ^]^ Metabolic reprogramming allows cancer cells to produce building blocks for biosynthesis, maintaining rapid proliferation even in harsh conditions.^[^ ^3^ ^]^ Glutamine is a central amino acid in cancer metabolism. Glutamine serves as a carbon donor for replenishing intermediates to tricarboxylic acid (TCA) cycle and as a nitrogen donor for biosynthesis of nucleotides and amino acids.^[^ ^4^, ^5^, ^6^ ^]^ Glutamine synthetase (GS), encoded by *GLUL*, is the only enzyme catalyzing de novo glutamine synthesis. GS is elevated in several cancers and is closely related to tumorigenesis.^[^ ^7^, ^8^, ^9^ ^]^ In GBM, GS sustains tumor growth under glutamine starvation by supplying nucleotides.^[^ ^10^, ^11^ ^]^ These findings point to an imperative role of GS in cancers, especially under conditions of limited glutamine. Previously, Nguyen et al. reported that GS stability is regulated by post‐translational modification (PTM) in eukaryotic cells.^[^ ^12^ ^]^ Acetylated GS is recognized and ubiquitinated, which undergoes proteasomal degradation in response to a high level of glutamine. Conversely, deacetylated GS becomes stabilized under glutamine‐starved conditions. Nevertheless, the detailed mechanism of GS regulation in GBM still remains ambiguous.

Acetylation is an important post‐translational modification (PTM) involved in multiple cellular processes. Histone deacetylases (HDACs) are epigenetic erasers that remove acetylation on lysine residues on not only histones but also non‐histone proteins. Unlike the other HDACs, HDAC6 predominantly localizes in the cytoplasm, enabling it to interact with multiple non‐histone proteins. HDAC6 regulates its target proteins by modulating their protein–protein interactions, stability, cell signaling, and subcellular localization. HDAC6 plays a crucial role in cancer by regulating proteins related to cancer progression.^[^ ^13^, ^14^, ^15^ ^]^ However, it remains unknown which HDAC enzyme modulates GS and whether it regulates glutamine metabolism in GBM.

Deubiquitinating enzymes (DUBs) are proteases that cleave ubiquitin linkages conjugated to lysine residues. DUBs maintain cellular homeostasis by balancing protein turnover and cell signaling in various contexts. Dysregulation of DUBs is intricately associated with tumorigenesis by modulating the stability of various tumor suppressors and oncogenes.^[^ ^16^, ^17^, ^18^ ^]^ Emerging evidence suggests that ubiquitin specific peptidase 9, X‐linked (USP9X), a crucial member of the USP family, plays an oncogenic role in GBM.^[^ ^19^, ^20^ ^]^ However, it is not clear whether USP9X controls cancer metabolism of GBM cells.

Herein, we aim to unravel the underlying mechanisms of GS homeostasis and its implications in GBM. We identify HDAC6 and USP9X as upstream regulators of GS in GBM. Mechanistically, deacetylation of GS mediated by HDAC6 facilitates USP9X to deubiquitinate GS, eventually stabilizing GS. Silencing of USP9X and HDAC6 suppresses proliferation of GBM in cell culture and animal models by reducing nucleotide availability and restricting stem‐like properties. Together, these results suggest that metabolic perturbation by targeting HDAC6 and USP9X could be a new therapeutic approach for GBM patients with high GS expression.

## 2 Results

### 2.1 HDAC6 Is a GS Interacting Partner That Regulates GS Protein Expression

**Figure 1:**
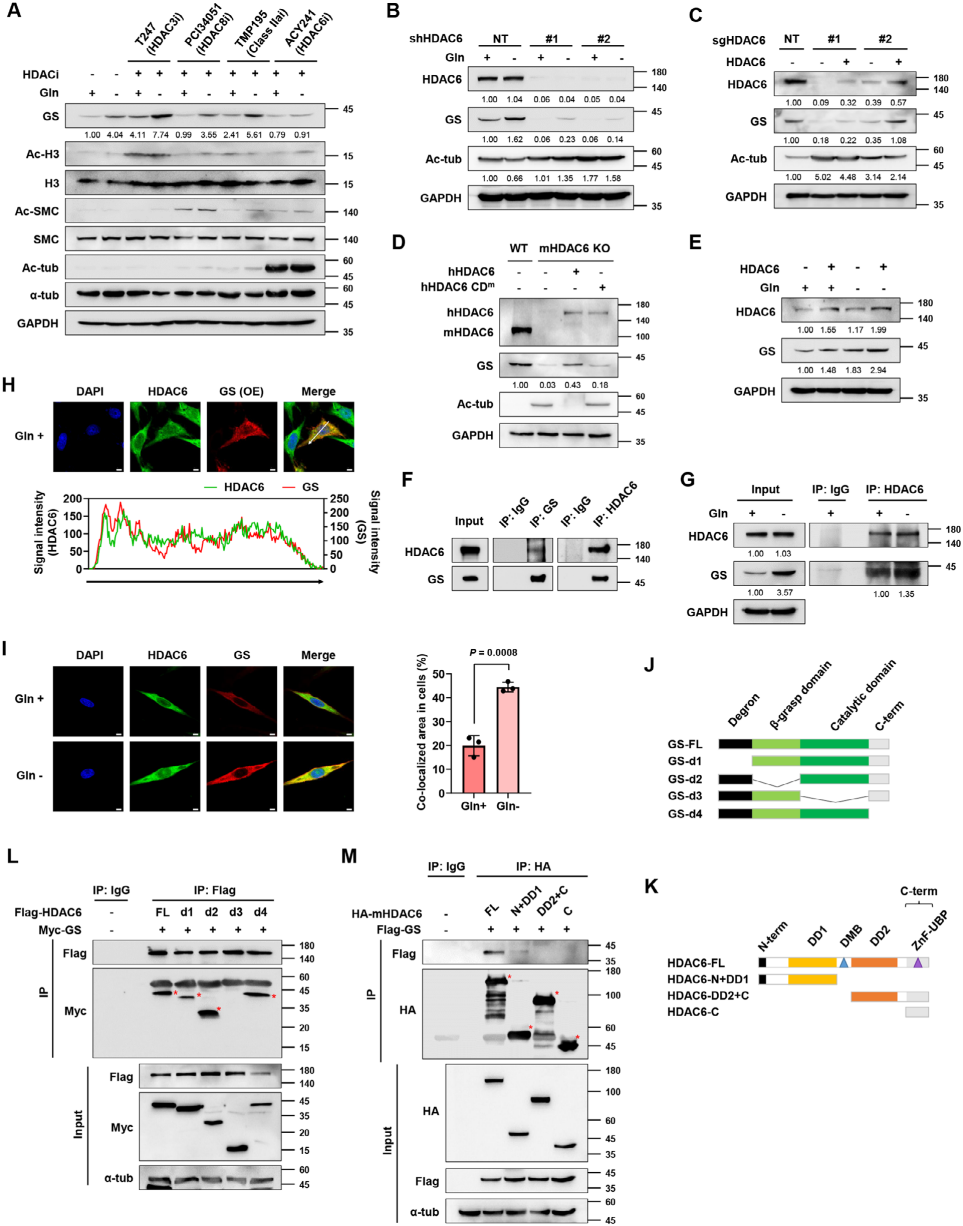
HDAC6 is a GS interacting partner that regulates GS protein expression. A) HDAC inhibitor screening with 10 µm PCI34051 and 2 µm T247, TMP195, and ACY241 in U251 cells. Cells were incubated with the indicated HDAC inhibitors for 24 h. B) IB analysis of GS expression in HDAC6 knockdown U251 cells incubated in Gln ± media for 24 h. C) Effects of HDAC6 reintroduction on GS expression in HDAC6 knockout U251 cells. D) IB analysis showing the GS expression in human HDAC6 WT (hHDAC6)‐ or catalytically inactive mutant (hHDAC6 CD^m^)‐introduced HDAC6 knockout MEF cells. E) Effects of HDAC6 overexpression in U251 cells incubated in Gln ± media for 24 h. F) IP analysis showing the interaction between HDAC6 and GS in HEK293T cells. G) IP analysis showing the interaction between HDAC6 and GS under glutamine depletion in U251 cells. Cells were incubated in Gln ± media for 24 h. H) Representative immunofluorescence images and signal intensity profiles across the line in the merged image showing co‐localization of HDAC6 and ectopically expressed GS in U251 cells. Scale bar, 5 µm. I) Representative immunofluorescence images and quantitative graph showing co‐localization of HDAC6 and GS in U251 cells incubated in Gln ± media for 48 h. Scale bar, 5 µm. Schematic diagrams of J) GS deletion constructs and K) HDAC6 domain constructs. L) IP analysis showing the GS interaction domain binding to HDAC6 in HEK293T cells. Red‐colored asterisks indicate co‐immunoprecipitated protein bands. M) IP analysis showing the HDAC6 interaction domain binding to GS in HEK293T cells. Red‐colored asterisks indicate immunoprecipitated protein bands.

Given that both bioinformatics analyses and cell‐based experiments showed that GS might be a promising target for GBM treatment, consistent with previous reports (Figures S1 and S2, Supporting Information),^[^ ^11^, ^21^, ^22^, ^23^, ^24^, ^25^, ^26^, ^27^ ^]^ we aimed to identify upstream regulators involved in GS regulation in GBM. To find deacetylases acting on GS, we treated U251 cells with several HDAC inhibitors. Of the HDAC inhibitors tested, HDAC6‐selective inhibitors prominently reduced GS protein expression in both glutamine‐replete and ‐depleted conditions (**Figure** **1**A; Figure S3A,B, Supporting Information). These observations led us to investigate the involvement of HDAC6 in GS regulation. Consistent with pharmacological inhibition, silencing HDAC6 using short‐hairpin RNA (shRNA) and CRISPR‐Cas9 significantly decreased GS protein levels compared to the non‐targeting (NT) controls in U251 cells (Figure S3D,E, Supporting Information) and various other cancer cells, including HCT116 (colorectal cancer), PC3 (prostate cancer), MM.1s (multiple myeloma), and SKOV3 (ovarian cancer) (Figure S3F–I, Supporting Information). Furthermore, HDAC6 silencing diminished glutamine starvation‐mediated GS induction (Figure 1B). In line with the changes in GS expression, HDAC6 knockout reduced GS activity (Figure S3J, Supporting Information). In addition, we found that reintroducing HDAC6 rescued GS protein level (Figure 1C), whereas catalytically dead mutant of HDAC6 could not restore the GS in HDAC6 knockout cells (Figure 1D), indicating that HDAC6's modulation of GS depends on its catalytic activity. Consistently, HDAC6 overexpression increased GS protein level (Figure 1E).

To test whether HDAC6 interacts with GS, we performed co‐immunoprecipitation and found that HDAC6 binds to GS (Figure 1F), and their interaction was reinforced when glutamine was depleted (Figure 1G; Figure S3K, Supporting Information). These results were also confirmed by immunofluorescence in U251 and U87 GBM cells (Figure 1H,I; Figure S3L,M, Supporting Information). Next, we sought to identify the interacting domains of HDAC6 and GS. We generated deletion constructs of GS (Figure 1J) and domain constructs of HDAC6 that include its catalytic deacetylase domains (DD1 and DD2) and zinc finger ubiquitin‐binding domain (ZnF‐UBP) (Figure 1K). We found that the catalytic domain of GS interacts with DD1 catalytic domain of HDAC6 (Figure 1L,M; Figure S3N, Supporting Information). These data suggest that HDAC6 interacts with GS and that HDAC6 activity sustains GS protein level.

### 2.2 Increased HDAC6 Activity upon Glutamine Starvation Supports GS Stability by Deacetylating GS

**Figure 2:**
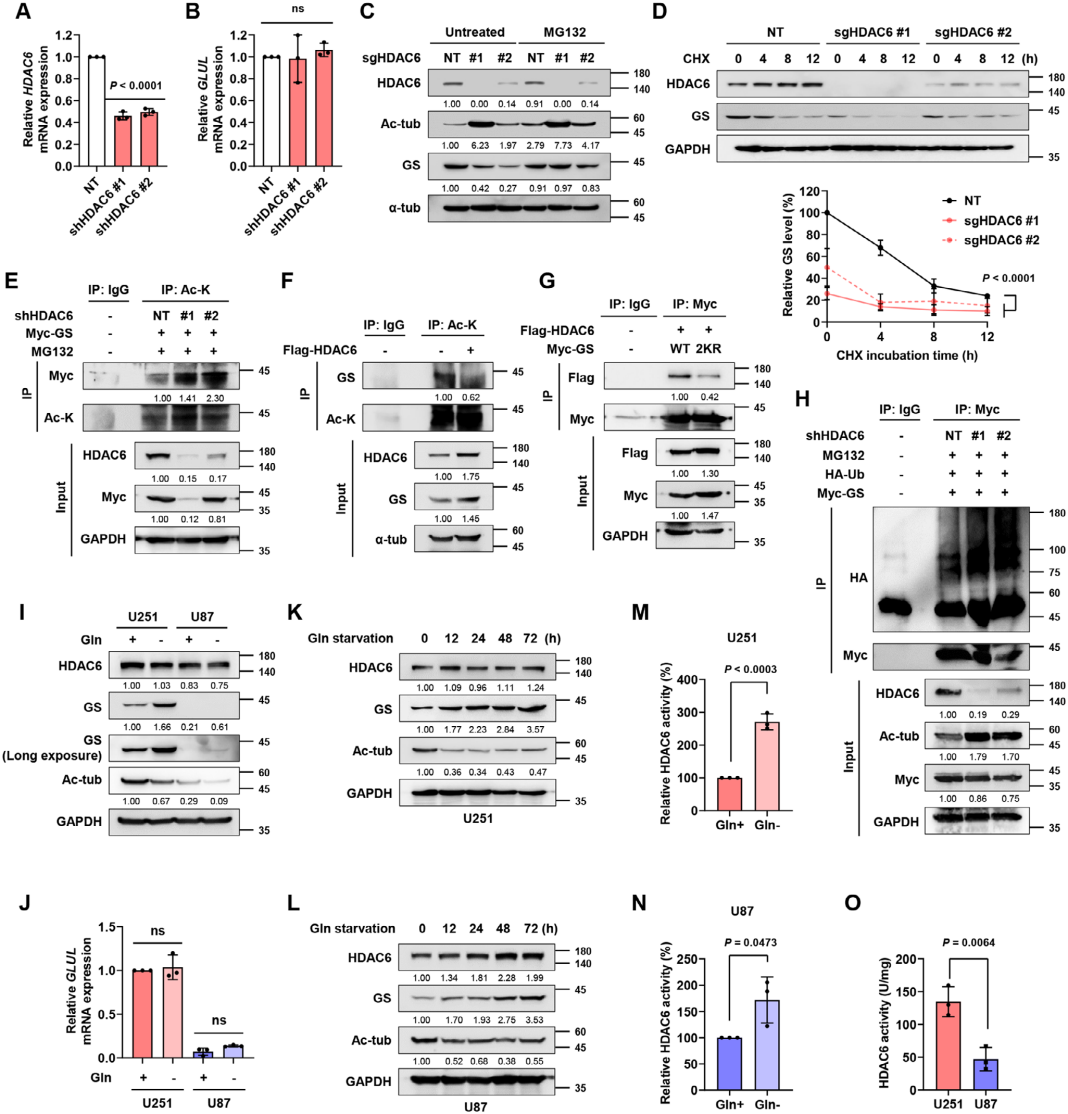
Increased HDAC6 activity upon glutamine starvation supports GS stability by deacetylating GS. mRNA levels of A) *HDAC6* and B) *GLUL* analyzed with qRT‐PCR upon knockdown of HDAC6 in U251 cells. C) IB analysis showing the effect of MG132 (20 µm, 4 h) on HDAC6 knockout U251 cells. D) IB analysis and quantification graph showing the effect of CHX (100 µm) on HDAC6 knockout U251 cells. E) IP analysis showing acetylation of GS in HDAC6 knockdown U251 cells. Cells were incubated with 20 µm MG132 for 4 h before making cell lysate. F) IP analysis showing acetylation of GS in HDAC6‐overexpressing U251 cells. G) IP analysis showing the interaction between HDAC6 and either WT or 2KR mutant of GS in U251 cells. H) IP analysis showing the ubiquitination of GS in HDAC6 knockdown U251 cells. I) IB analysis showing HDAC6, Ac‐tub, and GS under glutamine starvation in U251 and U87 cells. Cells were incubated in Gln ± media for 24 h. J) *GLUL* mRNA levels analyzed with qRT‐PCR under glutamine starvation in U251 and U87 cells. Cells were incubated in Gln ± media for 24 h. IB analysis showing HDAC6, Ac‐tub, and GS levels under glutamine starvation for various time points in K) U251 and L) U87 cells. Relative HDAC6 activity in glutamine‐depleted condition relative to that in glutamine‐replete condition in M) U251 and N) U87 cells. Cells were incubated in Gln ± media for 24 h. O) HDAC6 activity of U251 and U87 cells. Data are shown as mean ± SD. ns, not significant; *n* = 3.

Since HDAC6 regulates its targets at both transcriptional and post‐translational levels, we examined the change in GS (*GLUL*) mRNA levels. The GS mRNA levels were not altered by HDAC6 knockdown or knockout (**Figure** **2**A,B; Figure S3N,O, Supporting Information). Given that HDAC6 did not affect GS transcription, we next treated cells with MG132, a proteasome inhibitor. MG132 reversed the HDAC6 knockout‐induced GS reduction (Figure 2C), demonstrating that HDAC6 regulates GS through the proteasomal degradation pathway. To analyze protein homeostasis kinetics, we performed a cycloheximide (CHX) chase assay and found that HDAC6 depletion significantly reduced GS protein stability (Figure 2D). Next, we investigated whether HDAC6 deacetylates GS. Co‐immunoprecipitation results showed that GS acetylation increased with HDAC6 knockdown and decreased with HDAC6 overexpression (Figure 2E,F). To investigate whether the acetylation state of GS affects its interaction with HDAC6, we generated a GS‐2KR mutant, which mimics the deacetylation of previously reported acetylation sites, lysine (K) 11 and K14.^[^ ^12^ ^]^ The interaction between GS‐2KR mutant and HDAC6 was weaker than that of GS‐wild type (WT), demonstrating that acetylation of GS enhances its binding to HDAC6 (Figure 2G). Moreover, HDAC6 knockdown increased the ubiquitination of GS in GBM cells (Figure 2H). Together, these results show that HDAC6 increases GS stability by deacetylating GS.

Considering that GS increases under glutamine deprivation, we explored whether HDAC6 is involved in this phenomenon. Glutamine starvation upregulated GS protein (Figure 2I), but not mRNA (Figure 2J), indicating that GS induction by glutamine deprivation is not regulated at the transcriptional level. Interestingly, acetylation of tubulin, a well‐known substrate of HDAC6, decreased upon glutamine deprivation without alteration of HDAC6 protein levels (Figure 2I). These results led us to hypothesize that the enzymatic activity of HDAC6 might be elevated under glutamine deprivation. As expected, acetylation of tubulin was reduced in a time‐dependent manner, and HDAC6 activity increased upon glutamine starvation in both GBM cells (Figure 2K–N). These results demonstrate that elevated HDAC6 activity upregulates GS protein expression under glutamine starvation. To further understand the differences in GS protein expression patterns between GS‐high U251 and GS‐low U87 cells, we compared the HDAC6 activity in these cells. Indeed, HDAC6 activity was significantly higher in U251 than in U87 cells, correlating with their GS protein levels (Figure 2O). Based on these data, we suggest that increased HDAC6 activity upon glutamine deprivation is crucial for modulating GS protein expression.

### 2.3 USP9X Maintains GS Protein Stability by Binding to GS

**Figure 3:**
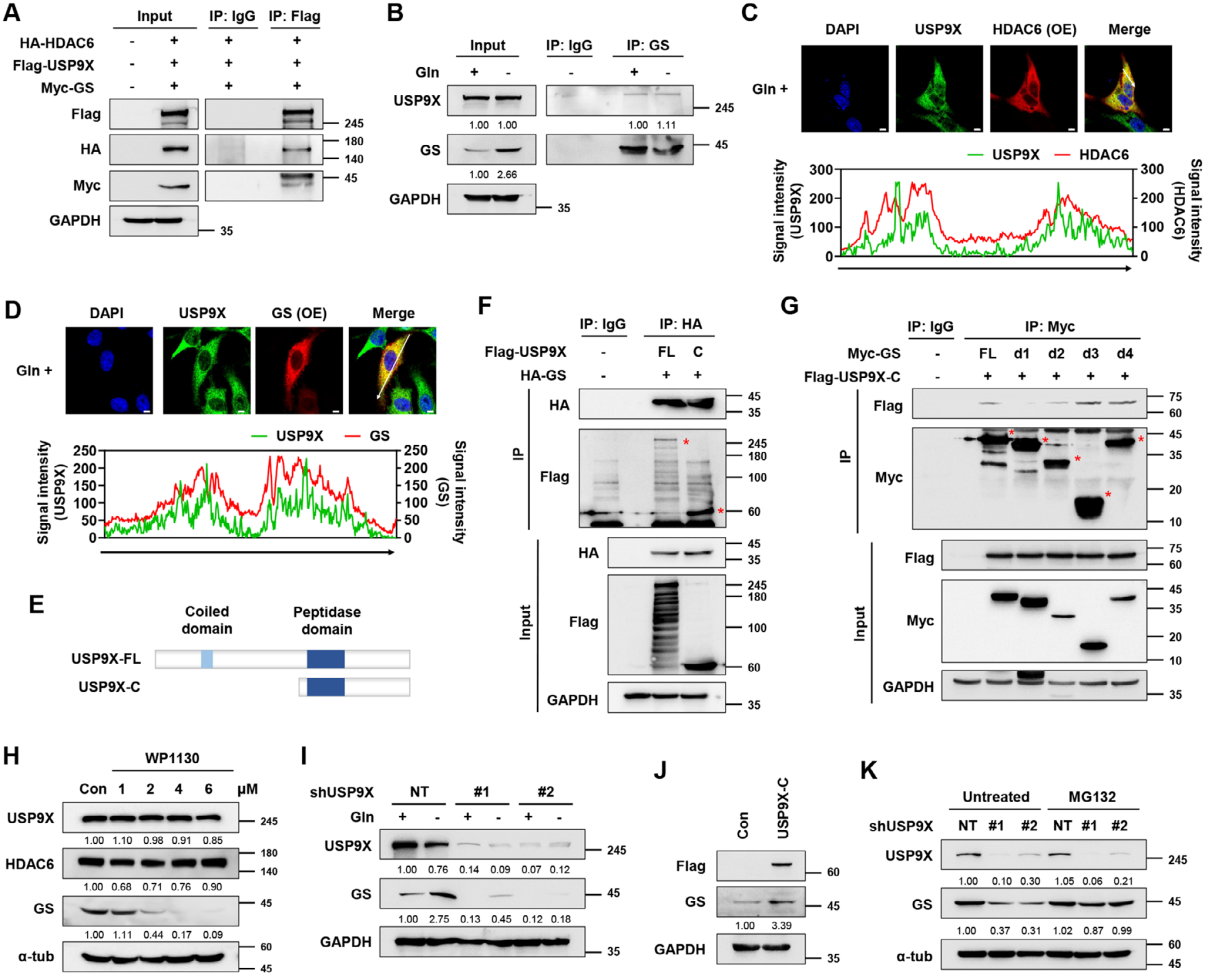
USP9X maintains GS protein stability by binding to GS. A) IP analysis showing the interaction between USP9X, HDAC6, and GS in HEK293T cells. B) IP analysis showing the interaction between USP9X and GS under glutamine depletion in U251 cells. Cells were incubated in Gln ± media for 24 h. Representative immunofluorescence images and signal intensity profiles across the line in the merged image showing C) co‐localization of USP9X and HDAC6 in HDAC6‐overexpressing U251 cells and D) co‐localization of USP9X and GS in GS‐overexpressing U251 cells. Scale bar, 5 µm. E) Schematic diagram of USP9X mutant constructs. F) IP analysis showing the USP9X interaction domain binding to GS in HEK293T cells. Red‐colored asterisks indicate co‐immunoprecipitated protein bands. G) IP analysis showing the interaction between GS domain binding to USP9X in HEK293T cells. Red‐colored asterisks indicate immunoprecipitated protein bands. H) IB analysis showing the effect of WP1130 on GS expression in U251 cells. Cells were incubated with WP1130 for 24 h. I) IB analysis of GS expression in USP9X knockdown U251 cells. Cells were incubated in Gln ± media for 24 h. J) Effect of USP9X overexpression on GS expression in U251 cells. K) IB analysis showing the effect of MG132 (20 µm, 4 h) on USP9X knockdown U251 cells.

To identify the interacting partners of HDAC6, we conducted mass spectrometry analysis after HDAC6 pull‐down and found that HDAC6 interacts with USP9X, a DUB enzyme (Figure S4A and Table S1, Supporting Information). However, USP9X did not alter the protein or ubiquitination levels of HDAC6 (Figure S4B, Supporting Information). We hypothesized that USP9X may form a complex with HDAC6 to regulate GS. Indeed, USP9X interacts with both HDAC6 and GS (**Figure** **3**A; Figure S4C, Supporting Information). Interestingly, the interaction between USP9X and GS did not change upon glutamine depletion (Figure 3B) while that of HDAC6 and GS was reinforced (Figure 1G). The co‐localization of USP9X, HDAC6, and GS was also validated with immunofluorescence (Figure 3C,D). Additionally, we found that peptidase domain‐containing C‐terminal of USP9X (USP9X‐C) interacts with degron of GS (Figures 1J and 3E–G).

Next, we analyzed the effect of pharmacological inhibition of USP9X on GS. Treatment with WP1130, a USP9X inhibitor, decreased GS in a dose‐dependent manner (Figure 3H). Genetic ablation of USP9X markedly reduced GS protein levels in U251 and U343 GBM cell lines as well as in SKOV3, an ovarian cancer cell line (Figure 3I; Figure S4D,E, Supporting Information). Consistently, USP9X knockdown decreased GS activity in U251 cells (Figure S4F, Supporting Information). Conversely, USP9X overexpression upregulated GS (Figure 3J; Figure S4H, Supporting Information). Since USP9X participates in the ubiquitin‐proteasome system, we checked if blocking proteasomal degradation could recover GS stability. Indeed, MG132 treatment rescued the downregulated GS protein upon USP9X knockdown (Figure 3K). These data imply that USP9X maintains GS stability by protecting it from proteasomal degradation.

### 2.4 USP9X Removes K48‐Linked Polyubiquitination on K91 and K103 of GS

**Figure 4:**
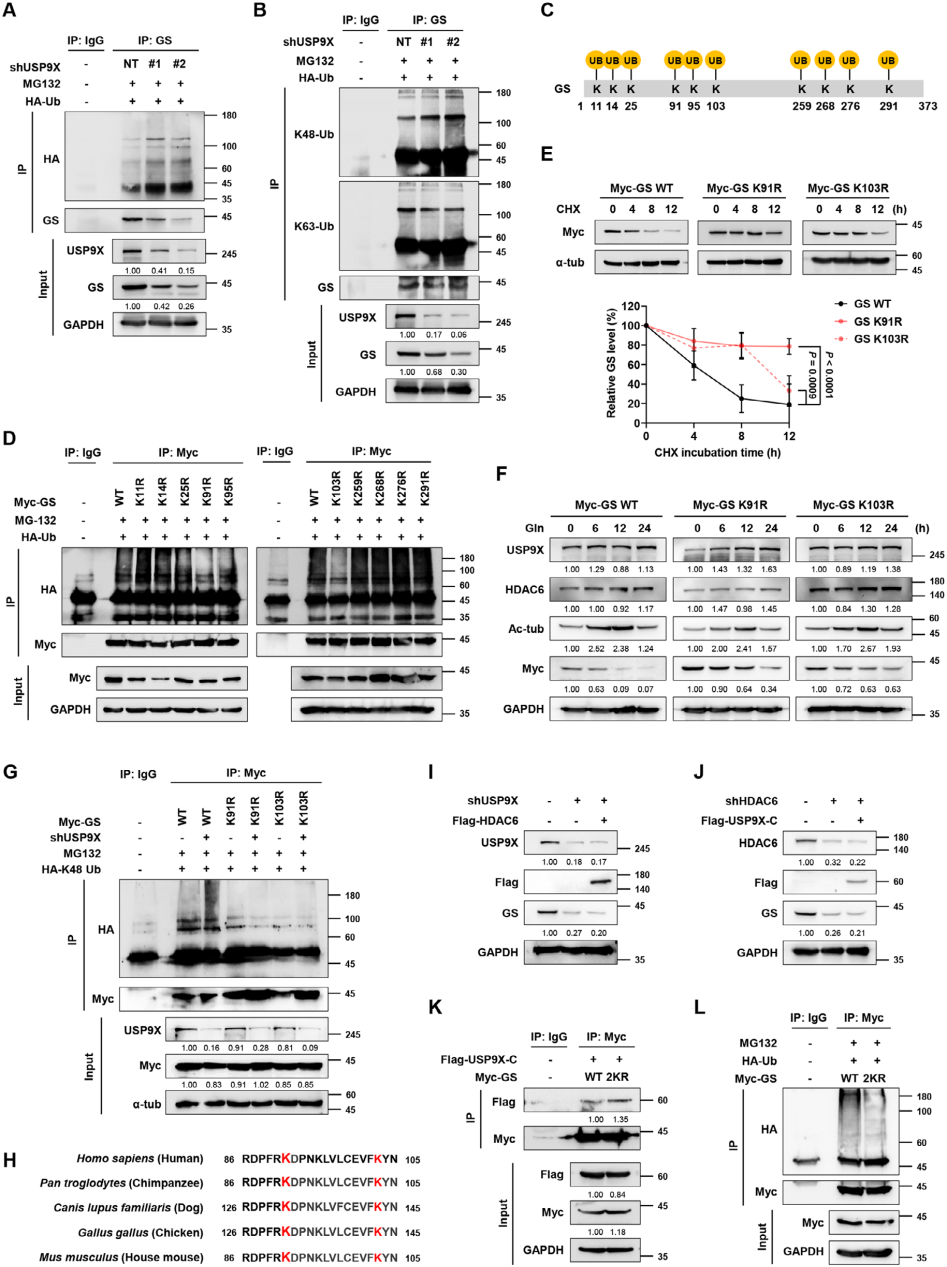
USP9X removes K48‐linked polyubiquitination on K91 and K103 of GS. A) Ubiquitination assay showing GS ubiquitination in USP9X knockdown U251 cells. B) Ubiquitination assay showing GS ubiquitination in USP9X knockdown U251 cells, detected with primary antibodies recognizing K48‐ and K63‐linked ubiquitin. C) Schematic diagram of the potential ubiquitination sites of GS. D) Ubiquitination assay showing the ubiquitination of GS mutants in U251 cells. E) CHX chase assay and quantification graph showing the protein stability of GS‐WT, ‐K91R, and ‐K103R. F) IB analysis showing the GS mutants after treatment with 4 mm glutamine following 48 h of glutamine deprivation. G) Ubiquitination assay showing the ubiquitination of GS‐WT, ‐K91R, and ‐K103R in USP9X knockdown U251 cells. H) Conservation of GS‐K91 and K103 across various organisms. I) IB analysis showing the effect of HDAC6 overexpression on USP9X knockdown U251 cells. J) IB analysis showing the effect of USP9X‐C overexpression on HDAC6 knockdown U251 cells. K) IP analysis showing the interaction between USP9X‐C and GS‐2KR in U251 cells. L) Ubiquitination assay showing GS‐2KR ubiquitination in U251 cells. Data are shown as mean ± SD, *n* = 3.

To further understand the process of GS degradation regulated by USP9X, we performed ubiquitination assays in various settings. USP9X knockdown increased GS ubiquitination while USP9X overexpression decreased GS ubiquitination, indicating that USP9X deubiquitinates GS (**Figure** **4**A; Figure S4I, Supporting Information). To determine the ubiquitin (Ub) linkage type involved in USP9X‐mediated GS deubiquitination, we conducted ubiquitination assay using antibodies that specifically recognize K48‐ or K63‐linked Ub (Figure 4B) and DNA constructs of K48‐ or K63‐linked Ub (Figure S4J, Supporting Information). These results showed that USP9X deubiquitinates K48‐linked Ub. Notably, K48‐linked polyubiquitination prompts proteasome‐mediated degradation while K63‐linked polyubiquitination regulates signal transduction.^[^ ^28^ ^]^ Together, these results demonstrate that USP9X prevents proteasome‐mediated degradation of GS.

To identify the ubiquitination sites of GS, we made several lysine‐to‐arginine mutants of potential ubiquitination sites obtained from Ubinet (Figure 4C).^[^ ^29^ ^]^ Among these, the ubiquitination levels of GS‐K91R and GS‐K103R significantly decreased compared to those of GS‐WT (Figure 4D). Consistently, K48‐linked ubiquitination of both mutants decreased, whereas their K63‐linked ubiquitination did not change (Figure S4K,L, Supporting Information). Notably, GS‐K91R and GS‐K103R mutants exhibited greater stability than GS‐WT in CHX assay (Figure 4E) and in glutamine‐refed cells (Figure 4F). Further investigation revealed that the ubiquitination of wild‐type GS increased in USP9X knockdown cells, whereas the ubiquitination of K91R and K103R GS mutants remained unchanged under the same conditions (Figure 4G). These results underscore that USP9X specifically removes K48‐linked polyubiquitination of GS at K91 and K103, thereby maintaining GS stability. Moreover, K91 and K103 of GS are conserved in organisms, implying that both sites are crucial for GS regulation (Figure 4H).

We then investigated the association between HDAC6 and USP9X in GS regulation. In the absence of USP9X, HDAC6 overexpression failed to rescue GS protein (Figure 4I), unlike in its presence (Figure 1E). Interestingly, GS protein level also did not change even when USP9X was ectopically expressed in HDAC6 knockdown cells (Figure 4J). These results suggest that cooperation of HDAC6 and USP9X is indispensable for regulation of GS stability. Indeed, the interaction between deacetylated GS (GS‐2KR) and USP9X‐C increased compared to that of GS‐WT (Figure 4K), and the ubiquitination of GS‐2KR decreased compared to that of GS‐WT (Figure 4L). These findings highlight the critical interplay between HDAC6 and USP9X in regulating GS stability.

### 2.5 Inhibition of HDAC6 and USP9X Suppresses GBM Cell Growth and Viability

**Figure 5:**
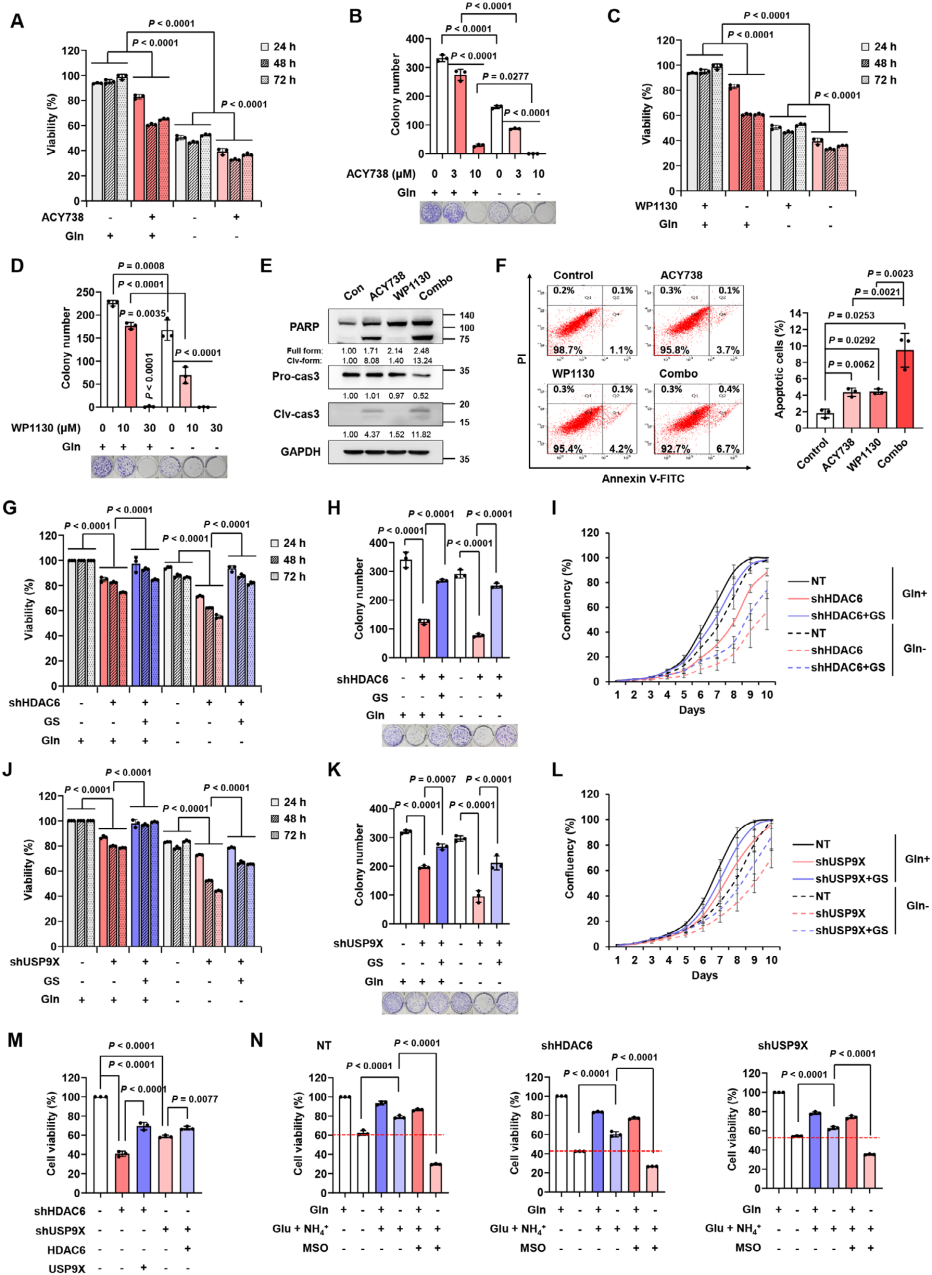
Inhibition of HDAC6 and USP9X suppresses GBM cell growth and viability. A) Cell viability and B) colony formation analyses showing the effects of ACY738 (5 µm) in U251 cells incubated in Gln ± media. C) Cell viability and D) colony formation analyses showing the effects of WP1130 (5 µm) in U251 cells incubated in Gln ± media. E) IB analysis showing apoptosis induction by 5 µm ACY738, WP1130, and the combination of both inhibitors in U251 cells. Cells were incubated with the inhibitors for 24 h. F) Apoptosis assay with Annexin V/PI staining after treatment with 5 µm ACY738, WP1130, and the combination of both inhibitors. Apoptotic cells (%) were then calculated. G) Cell viability, H) colony formation, and I) cell proliferation analyses (*n* = 5) showing the effects of HDAC6 knockdown and the rescue effects of GS overexpression in U251 cells incubated in Gln+ (represented by solid lines)/Gln− (represented by dotted lines) media containing 4 mm of Glu and 0.8 mm of NH_4_Cl. J) Cell viability, K) colony formation, and L) cell proliferation analyses (*n* = 5) showing the effects of USP9X knockdown and the rescue effects of GS overexpression in U251 cells incubated in Gln+ (represented by solid lines)/Gln− (represented by dotted lines) media containing 4 mm of Glu and 0.8 mm of NH_4_Cl. M) Cell viability of USP9X overexpressed HDAC6 knockdown U251 cells and HDAC6 overexpressed USP9X knockdown U251 cells. Cells were incubated in Gln+ media supplemented with 4 mm Glu, and 0.8 mm NH_4_Cl, and cell viability was measured 72 h after seeding. N) Cell viability of HDAC6 knockdown and USP9X knockdown U251 cells incubated in Gln ± media supplemented with or without 4 mm Glu, 0.8 mm NH_4_Cl, and 1 mm MSO for 72 h. Data are shown as mean ± SD; *n* = 3, unless otherwise noted.

Since HDAC6 and USP9X stabilize GS protein, we next investigated the effect of HDAC6 and USP9X ablation on GBM cell growth. Silencing HDAC6 abolished cell growth and colony formation (Figure S5A,B, Supporting Information), while HDAC6 overexpression increased GBM colony formation (Figure S5C, Supporting Information). Similar to HDAC6 knockdown, USP9X knockdown repressed GBM cell growth and colony formation (Figure S5D,E, Supporting Information). Pharmacological inhibition of HDAC6 and USP9X using ACY738, a blood‐brain barrier‐permeable HDAC6 inhibitor, and WP1130 also suppressed GBM cell growth and induced apoptosis (**Figure** **5**A–F) at concentrations that did not affect normal cell viability (Figure S6, Supporting Information).

The introduction of GS reversed the attenuated cell viability, cell growth, and colony formation of HDAC6 and USP9X knockdown GBM cells in both glutamine‐replete and ‐depleted conditions (Figure 5G–L and Figure S5F, Supporting Information), indicating that targeting HDAC6 and USP9X suppresses GBM cell growth by modulating GS protein. Surprisingly, cell viability was slightly recovered when HDAC6 knockdown cells and USP9X knockdown cells were overexpressed with one another (Figure 5M) even though GS protein levels did not change (Figure 4I,J). This observation is consistent with previously reported studies that HDAC6 and USP9X exhibit various oncogenic roles other than GS regulation in GBM.^[^ ^19^, ^20^, ^30^, ^31^ ^]^

Supplementation of glutamate and ammonia under glutamine deprivation restored cell viability in control cells, but to a lesser extent in HDAC6 and USP9X knockdown cells (control cells; ≈87%, HDAC6 knockdown cells; ≈77%, and USP9X knockdown cells; ≈74%) (Figure 5N). Additionally, methionine sulfoximine (MSO) treatment hindered the rescue of cell growth upon supplementation of glutamate and ammonia, demonstrating that HDAC6 and USP9X maintain GBM cell growth by modulating GS activity.

### 2.6 Silencing of HDAC6 and USP9X Attenuates GBM Tumorigenesis In Vivo

**Figure 6:**
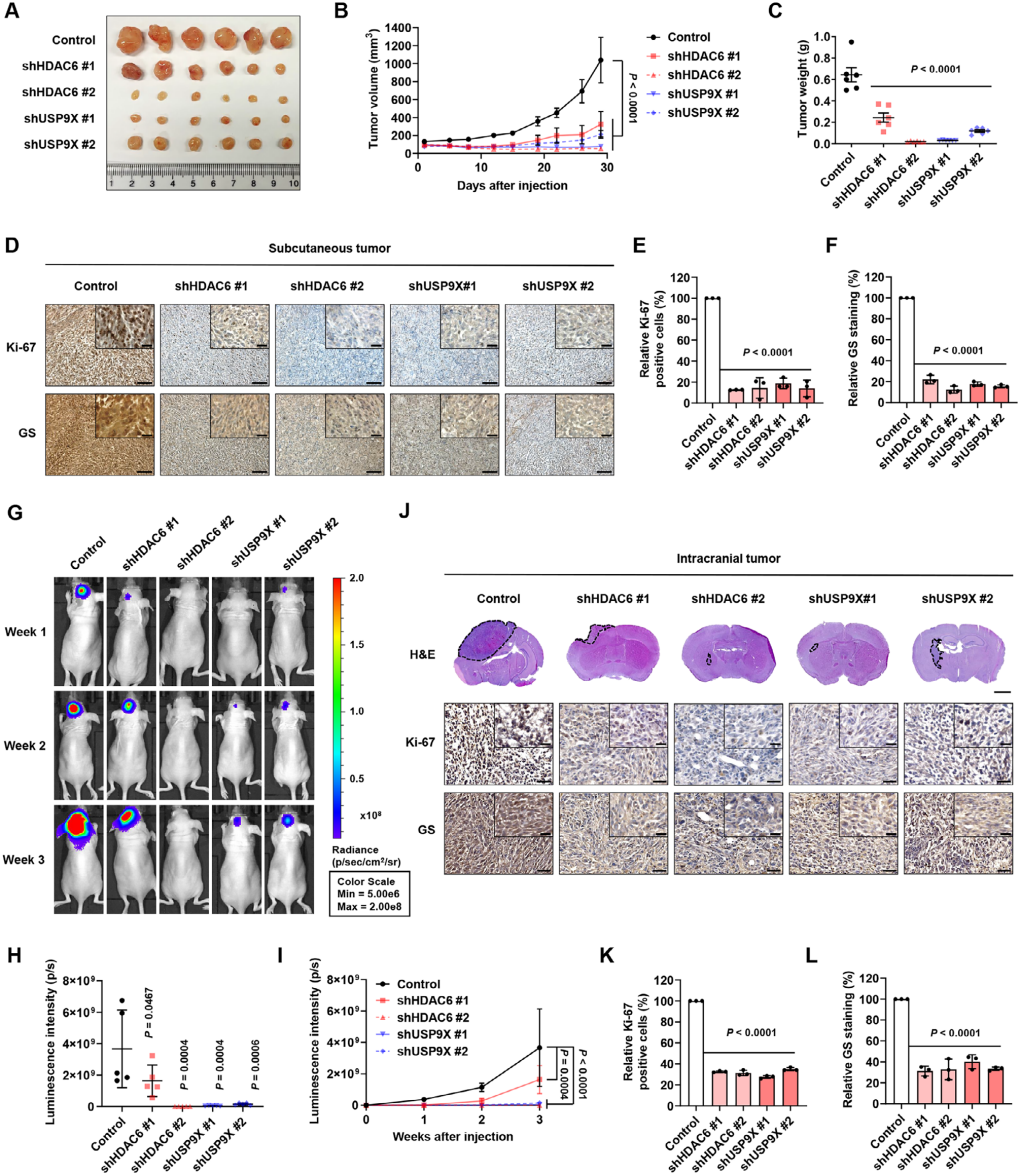
Silencing of HDAC6 and USP9X attenuates GBM tumorigenesis in vivo. A) Images, B) tumor volumes, and C) tumor weights of U251‐derived xenograft tumors (*n* = 6). D) Representative images for IHC staining for Ki‐67 and GS in subcutaneous tumors, and graph showing the relative number of E) Ki‐67 positive cells and F) GS staining intensity. Scale bar of low magnification, 100 µm; Scale bar of high magnification, 20 µm. G) Representative bioluminescence images of U251‐derived intracranial xenograft mice taken at weeks 1, 2, and 3 after cell implantation (*n* = 5). H) Luminescence intensity of U251‐derived intracranial xenograft tumors in week 3 and I) over 3 weeks (*n* = 5). J) Representative images for H&E and IHC staining in intracranial tumors, and graph showing the relative number of K) Ki‐67 positive cells and L) GS staining intensity. Scale bar of H&E staining, 2 mm, Scale bar of low magnification in IHC staining, 50 µm; Scale bar of high magnification in IHC staining, 20 µm. Data are shown as mean ± SD; *n* = 3, unless otherwise noted.

To validate the effects of HDAC6 and USP9X inhibition in animal models, we established a GBM subcutaneous xenograft model by implanting HDAC6 and USP9X‐silenced U251 cells into immunocompromised nude mice. Ablation of HDAC6 and USP9X substantially reduced tumor volume and weight in the GBM xenograft (**Figure** **6**A–C). Immunohistochemistry analysis further confirmed that HDAC6 and USP9X knockdown in subcutaneous tumors decreased the expression of GS and Ki‐67, a cell proliferation marker (Figure 6D–F). To better mimic physiological environment, these findings were validated using an intracranial xenograft model. Consistent with the subcutaneous model, knockdown of HDAC6 and USP9X notably suppressed GBM tumor growth (Figure 6G–I; Figure S7, Supporting Information) and reduced Ki‐67 and GS expression (Figure 6J–L) in the intracranial model. These results demonstrate that targeting HDAC6 and USP9X hampers GBM tumorigenesis in vivo, consistent with in vitro results.

### 2.7 Ablation of USP9X and HDAC6 Impairs De Novo Nucleotide Synthesis

**Figure 7:**
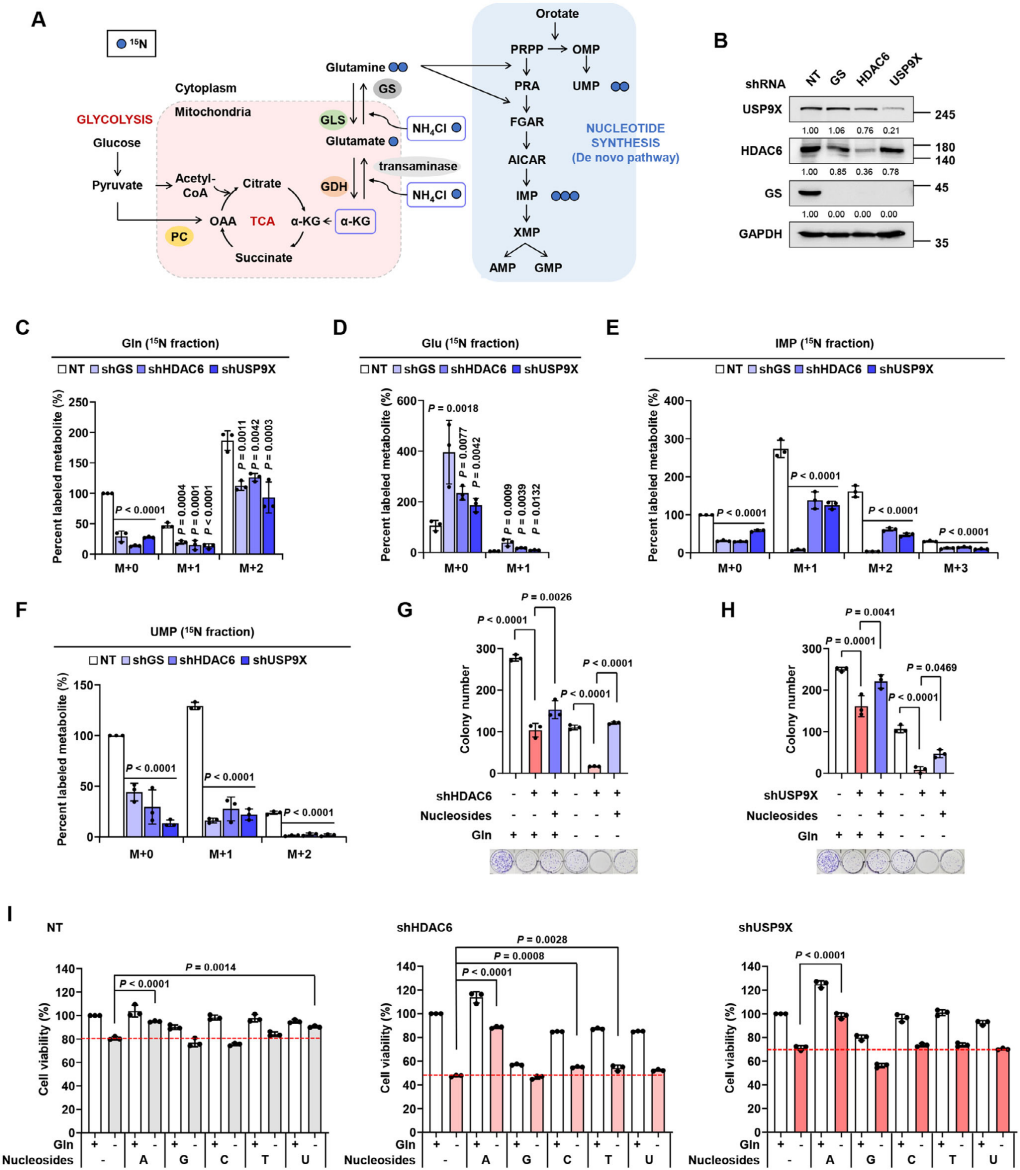
Ablation of USP9X and HDAC6 impairs de novo nucleotide synthesis. A) Schematic illustration of glutamine metabolism. B) IB analysis showing that GS, HDAC6, and USP9X are knockdown in U251 cells. Relative levels of C) ^15^N_2_‐NH_4_Cl‐derived Gln, D) Glu, E) IMP, and F) UMP normalized to control NT cells. Cells were glutamine‐depleted for 12 h before labeling and incubated in glutamine‐free media containing 6 mm dmKG and 0.8 mm ^15^N_2_‐NH_4_Cl for 24 h. MRM transition of metabolites is summarized in Table S2 (Supporting Information). Colony formation assays in G) HDAC6 knockdown and H) USP9X knockdown U251 cells. Cells were incubated in Gln ± media supplemented with a combination of A, G, C, T, and U (0.2 mm each). I) Cell viability analysis of HDAC6 knockdown and USP9X knockdown U251 cells. Cells were incubated in Gln ± media supplemented with 0.2 mm each A, G, C, T, and U for 72 h, as indicated. Data are shown as mean ± SD; *n* = 3.

Next, we investigated the metabolic fate of de novo synthesized glutamine (**Figure** **7**A) in HDAC6 and USP9X knockdown GBM cells and compared their effects to those of GS knockdown (Figure 7B). We traced the nitrogen of glutamine by supplying cells with ^15^N‐NH_4_Cl and dimethyl‐ketoglutarate (dmKG), a cell‐permeable form of α‐ketoglutarate (αKG), after 12 h of glutamine starvation. The ^15^N fraction of glutamine decreased in all fractions while that of glutamate increased (Figure 7C,D). Similar to the nitrogen‐tracing results, ^13^C‐labeled glutamate and αKG considerably accumulated in HDAC6 and USP9X knockdown cells (Figure S8, Supporting Information). In addition, silencing of HDAC6 and USP9X reduced glutamine levels and increased glutamate levels in U251‐derived subcutaneous xenograft tumors (Figure S9, Supporting Information). These results indicate that USP9X and HDAC6 knockdown suppress de novo glutamine synthesis. Since glutamine is vital for de novo nucleotide synthesis, we next examined the ^15^N fraction of inosine monophosphate (IMP) and uridine monophosphate (UMP), intermediates in purine synthesis and pyrimidine synthesis, respectively. We found that ^15^N labeling of both IMP and UMP decreased in HDAC6 and USP9X knockdown cells (Figure 7E,F), demonstrating that knockdown of HDAC6 and USP9X restrains nucleotide biosynthesis.

A previous report showed that nucleotide availability is decisive for GBM cell survival when glutamine is depleted.^[^ ^11^ ^]^ Thus, we investigated whether the growth inhibitory effects of HDAC6 and USP9X knockdown are associated with nucleotide availability. We treated the cells with nucleosides‐mixed solution, and found that nucleoside supplementation rescued the decreased colony‐forming capacity of HDAC6 and USP9X knockdown cells (Figure 7G,H). To further identify the nucleotide that plays a critical role in GBM growth when glutamine is depleted, we supplemented glutamine‐starved U251 cells with each nucleoside. The addition of adenosine most significantly rescued HDAC6 and USP9X knockdown cells, which were susceptible to glutamine deprivation (Figure 7I). Together, these data suggest that HDAC6 and USP9X sustain GBM cell growth by synthesizing nucleotides, especially adenosine derived from glutamine, highlighting the importance of HDAC6 and USP9X in glutamine metabolism of GBM.

### 2.8 Silencing of HDAC6 and USP9X Diminishes Stem Cell‐Like Properties of GBM Cells

GBM stem‐like cells (GSCs) are a subpopulation of GBM cells that display intrinsic chemoresistance and radioresistance.^[^ ^32^ ^]^ Since GS has been reported to be increased in cancer stem cells, including GSCs,^[^ ^11^, ^33^ ^]^ we analyzed gene expression of *GLUL*, *HDAC6*, and *USP9X* in GSCs using the Gene Expression Omnibus (GEO) dataset (GSE23806). *GLUL*, *HDAC6*, and *USP9X* were upregulated not only in GSCs but also in neurospheres and primary tumors compared to conventional cell lines (Figure S10A–D, Supporting Information). Additionally, in the same dataset, the gene expression of *GLUL* positively correlates with that of *HDAC6* and *USP9X* (Figure S10E,F, Supporting Information). These results led us to further explore whether HDAC6 and USP9X are associated with the stemness of GBM. We found that silencing HDAC6 and USP9X attenuates the sphere‐forming capacity of GBM cells (Figure S10G,H, Supporting Information). Furthermore, ablation of HDAC6 and USP9X reduced the gene expression of *Nanog* and *Sox2* as well as the protein levels of OCT4, SOX2, and Nanog, which are stemness markers (Figure S10I–K, Supporting Information). Collectively, targeting HDAC6 and USP9X diminishes the stem cell‐like properties of GBM.

### 2.9 HDAC6 and USP9X Positively Correlate with GS and Are Associated with Poor Prognosis in GBM

**Figure 8:**
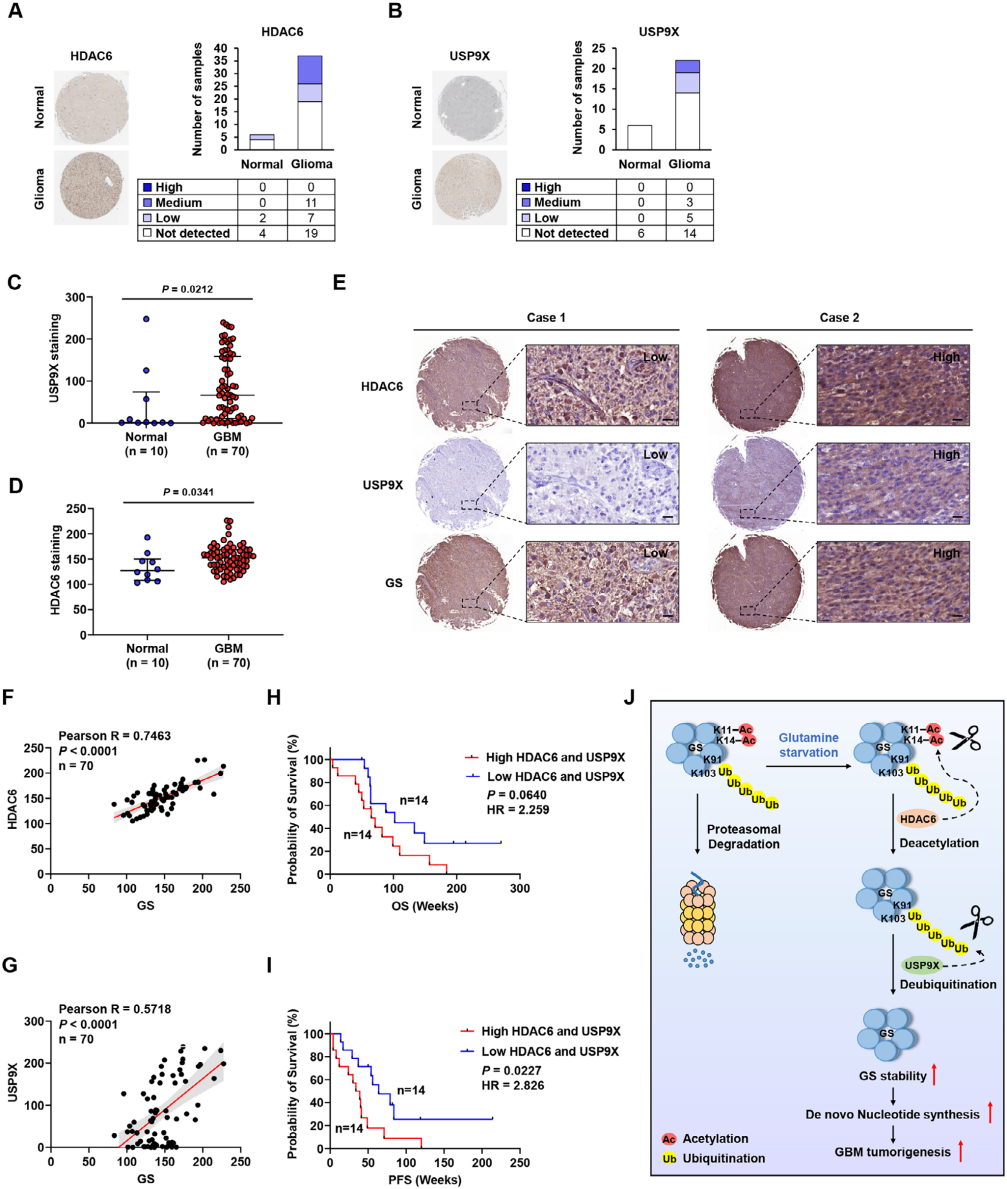
HDAC6 and USP9X positively correlate with GS and poor prognosis in GBM. Representative images and the number of stained samples from IHC staining of A) HDAC6 and B) USP9X in glioma and normal tissues. Data were obtained from the Human Protein Atlas database. IHC staining of C) HDAC6 and D) USP9X in TMA sections of GBM and normal tissues. E) Representative images of HDAC6, USP9X, and GS IHC staining in the GBM TMA sections. Scale bar, 20 µm. F–I) Correlation analysis between HDAC6, USP9X, and GS in the GBM TMA sections (*n* = 70). J,K) Kaplan–Meier survival analysis of primary GBM samples from the GEO dataset (GSE42669) stratified by HDAC6 and USP9X expression for overall survival (OS) and progression‐free survival (PFS). L) A Schematic diagram of HDAC6 and USP9X‐mediated GS regulation in GBM. Data are shown as mean ± SD.

We aimed to examine the clinical relevance of HDAC6 and USP9X in GBM. HDAC6 and USP9X protein levels were increased in glioma and GBM tissues compared to normal tissues in publicly available databases, including Human Protein Atlas^[^ ^34^ ^]^ (**Figure** **8**A,B). Similarly, we observed elevated HDAC6 and USP9X protein levels in a GBM tissue microarray (Figure 8C,D), and there was a positive correlation between HDAC6, USP9X, and GS in GBM tissues (Figure 8E–G). Furthermore, high HDAC6 and USP9X expression in GBM correlated with poor clinical outcomes (Figure 8H,I; Figure S11, Supporting Information). These data suggest that HDAC6 and USP9X could be druggable targets in GBM.

## 3 Discussion

Emerging evidence suggests that cancer cells utilize metabolic reprogramming to support increased energy demands. Rapidly proliferating cancer cells utilize glutamine as a carbon and nitrogen source to generate essential biomolecules. Indeed, previous studies report that glutamine anabolism is increased in GBM compared to the normal brain.^[^ ^10^, ^11^ ^]^ Here, our results show that GBM cells rewire the metabolic flux toward de novo nucleotide biosynthesis to promote cancer progression by HDAC6‐ and USP9X‐mediated GS stabilization (Figure 8J). Mechanistically, HDAC6 removes acetylation on GS, which then undergoes USP9X‐mediated deubiquitination. Interestingly, HDAC6 and USP9X cooperate to stabilize GS. USP9X deubiquitinates K48‐linked polyubiquitination on deacetylated GS. It is worthy of note that we discovered K91 and K103 as the most probable sites of ubiquitination that are engaged in proteasomal degradation. However, this study focuses on only ten lysine residues out of nineteen lysine residues on GS. Expanding the investigation to include other lysine residues could offer more comprehensive understanding of the dynamic regulatory mechanisms governing GS. Interestingly, Ling et al. recently demonstrated that SUMOylation at lysine 372 destabilizes GS by examining all lysine sites of GS.^[^ ^35^ ^]^ Small ubiquitin‐like modifier (SUMO), a member of the ubiquitin‐like (Ubl) proteins, regulates protein functions by attaching to lysine residues. This overlap in modification sites suggests that Ubl modifications may compete for the same lysine sites, potentially modifying the function of the target protein in distinct ways.^[^ ^36^ ^]^ Additionally, Ubls have been implicated in the activation of ubiquitin ligases, further influencing protein regulation. Therefore, exploring the interplay between ubiquitination and SUMOylation could provide valuable insights into the intricate mechanisms underlying the fine‐tuned regulation of GS.

A previous study showed that HDAC3 deacetylates and stabilizes GS through HDAC inhibitor screening (but this screening does not include HDAC6 inhibitors) and confirmed the result with genetic silencing in liver cancer cells.^[^ ^33^ ^]^ Similarly, we observed that T247, an HDAC3‐selective inhibitor, reduces GS protein levels in HepG2 cells (Figure S3C, Supporting Information). However, T247 failed to reduce GS protein levels in GBM cells; GS protein levels were rather increased (Figure 1A). Moreover, genetic ablation of HDAC6 decreases GS in not only GBM but also in other cancer cells (Figure S3D–I, Supporting Information). A recent paper reported USP15 as a DUB antagonizing ubiquitination of GS.^[^ ^37^ ^]^ However, in our study, USP15 knockout did not decrease GS protein in GBM cells (Figure S4G, Supporting Information), whereas genetic ablation of USP9X decreased GS in GBM and ovarian cancer (Figure 3I; Figure S4D,E, Supporting Information). These results indicate that HDACs and DUBs regulate their substrates context‐dependently, especially in different cancer types.

In vitro and in vivo studies show that targeting HDAC6 and USP9X remarkably represses GBM tumorigenesis and disturbs glutamine metabolism. The function of HDAC6 and USP9X in glutamine metabolism has been recognized for the first time in this study. Through metabolic isotope tracing, we identified that silencing HDAC6 and USP9X diminishes nucleotide availability. Furthermore, we demonstrated that nucleosides, especially adenosine, derived from glutamine are pivotal to meet the anabolic demands of rapidly growing GBM cells.

Apart from their role in regulating GS, both HDAC6 and USP9X have been reported to promote the malignant potential of GBM via various mechanisms. HDAC6 promotes GBM tumorigenesis by upregulating Sp1, a transcription factor that is responsible for protecting GBM cells from temozolomide^[^ ^30^ ^]^ and activating DNA damage response.^[^ ^38^, ^39^ ^]^ In addition, HDAC6 promotes GBM proliferation by regulating cilia formation^[^ ^31^ ^]^ and modulating lncRNA‐microRNA‐mRNA network.^[^ ^40^ ^]^ USP9X is deregulated in diverse cancers and either stimulates or suppresses cancer development in a context‐dependent manner.^[^ ^19^, ^41^, ^42^, ^43^, ^44^ ^]^ In GBM, USP9X is profoundly associated with tumorigenesis, controlling GBM cell survival and radio‐sensitivity by stabilizing Mcl‐1, a Bcl‐2 family anti‐apoptotic protein.^[^ ^19^ ^]^ Additionally, USP9X maintains mesenchymal features of GSC by stabilizing ALDH1A3, a key determinant of the mesenchymal identity of GSCs.^[^ ^20^ ^]^ Collectively, targeting HDAC6 and USP9X would be beneficial regardless of their roles in GS regulation.

By analyzing the human GBM tissue, we observed that HDAC6 and USP9X are increased in GBM, while GS is heterogeneously expressed. The heterogeneity of GS expression in GBM has been previously reported.^[^ ^11^ ^]^ GS protein levels vary among GBM patients, with about 30% exhibiting high GS expression and 20% exhibiting low GS expression, and the rest showing moderate expression.^[^ ^11^ ^]^ Therefore, targeting HDAC6 and USP9X might be a valuable therapeutic strategy for GBM patients with high GS expression.

GSCs are impediments to efficient GBM treatment. GSCs are resistant to conventional chemotherapy and radiotherapy, thus remaining GSCs after chemotherapy treatment eventually leads to tumor relapse.^[^ ^45^ ^]^ These findings clearly highlight the need for developing effective methods to eradicate GSCs to prevent GBM recurrence. In the present study, sphere‐forming capacity and stemness markers were reduced by genetic ablation of HDAC6 and USP9X. Therefore, we propose HDAC6 and USP9X as promising targets for overcoming the barriers posed by GSCs.

Pan‐HDAC inhibitors have been approved for the treatment of hematologic malignancies, and HDAC6‐selective inhibitors are actively being developed and undergoing clinical trials.^[^ ^46^, ^47^ ^]^ However, the development of USP9X‐selective inhibitors has been relatively underexplored, with existing compounds exhibiting limited selectivity. Targeting the HDAC6/USP9X/GS axis not only by inhibiting HDAC6 and USP9X, but also by disrupting their protein–protein interactions with GS could present a promising therapeutic approach.

Cancer metabolism consists of highly interconnected and intricately regulated pathways, often resulting in compensatory mechanisms.^[^ ^48^ ^]^ GBM cells with high GS expression synthesize glutamine for their own use, while those with low GS expression rely on glutamine acquisition from surrounding astrocytes.^[^ ^11^ ^]^ Consequently, targeting HDAC6 or USP9X to reduce GS expression may induce compensatory mechanisms such as upregulation of glutamine transporters. Additionally, given the diverse pathways involved in GS regulation,^[^ ^12^, ^21^, ^22^, ^35^, ^49^, ^50^, ^51^, ^52^, ^53^, ^54^, ^55^ ^]^ alternative mechanisms inducing GS expression may be activated. Therefore, further investigation elucidating these compensatory metabolic pathways when targeting HDAC6 and USP9X in clinical setting is critical for substantiating the translational applicability of these findings.

In conclusion, our data suggest that HDAC6 and USP9X are functional determinants of glutamine metabolism by regulating GS, ultimately affecting GBM survival. The current study uncovers the novel mechanism of regulating glutamine metabolism and presents a compelling idea for a new therapeutic strategy in GBM treatment.

## 4 Experimental Section

### Antibodies

The primary antibodies used in this study are as follows: anti‐GS (Santa Cruz Biotechnology, sc‐74430 and Genetex, GTX109121), anti‐Ac‐tub (Sigma‐Aldrich, T6793), anti‐α‐tub (Santa Cruz Biotechnology, sc‐32293), anti‐Ac‐H3 (Millipore, 06‐599), anti‐H3 (Millipore, 06‐755), anti‐Ac‐SMC (Millipore, MABE1073), anti‐SMC (Bethyl, A300‐060A‐M), anti‐HDAC6 (Bethyl, A301‐342A), anti‐DDDK‐tag (MBL Life Science, M185‐3L), anti‐Myc‐tag (MBL Life Science, M192‐3), anti‐HA‐tag (MBL Life Science, M180‐3), anti‐Ac‐K (Millipore, 05‐515 and Millipore, 06‐933), anti‐USP9X (Santa Cruz Biotechnology, sc‐365353), anti‐K48‐Ub (Cell Signaling Technology, #4289), anti‐K63‐Ub (Cell Signaling Technology, #5621), anti‐PARP (BD Pharmingen, #551024), anti‐caspase3 (Cell Signaling Technology, #9662), anti‐GAPDH (Genetex, GTX100118), and anti‐β‐actin (Santa Cruz Biotechnology, SC‐47778).

### Reagents

To inhibit HDAC activity, the following pan‐HDAC inhibitors were used: Trichostatin A (TSA) (Selleck Chemicals, S1045), SAHA (Selleck Chemicals, S1047), and LBH589 (Selleck Chemicals, S1030). To inhibit Class HDAC I activity, FK228 (Sigma‐Aldrich, SML1175) was used. To selectively inhibit HDAC3 and HDAC8, T247 (TCI Chemicals, A2897) and PCI34051 (Med Chem Express, HY‐15224) were used, respectively. To inhibit Class IIa HDAC, TMP195 (Med Chem Express, HY‐18361) was used. To selectively inhibit HDAC6, following HDAC6 inhibitors were used: A452 (purity 99%, a γ‐lactam based HDAC6 inhibitor, this chemical was kindly provided by Dr. Gyoonhee Han (Yonsei University, Seoul, Korea)), ACY241 (Selleck Chemicals, S8464), Tubastatin A (Selleck Chemicals, S8049), and CAY10603 (Selleck Chemicals, S7596). To inhibit SIRT1 and SIRT2, a SIRT inhibitor, Tenovin 6 (Selleck Chemicals, S4900) was used. To inhibit USP9X, WP1130 (Cayman Chemical, #15227) was used.

### DNA Constructs

For overexpression experiments, pcDNA3‐Flag‐HDAC6, pcDNA3‐HA‐HDAC6, pcDNA3‐Flag‐HDAC6 CD^m^, pcDNA3‐HA‐mHDAC6 (FL, N, N+DD1, DD2+C, C), pcDNA3‐Myc‐GS (FL, d1, d2, d3, d4) were constructed and used. pEZ‐M12‐Flag‐mUSP9X (FL, C) was kindly provided by Sehyoun Yoon (Northwestern University, Evanston, IL, USA).^[^ ^56^ ^]^ The mutant constructs for Myc‐tagged GS were generated using site‐directed mutagenesis (K11R, K14R, 2KR (K11R and K14R), K25R, K91R, K95R, K103R, K259R, K268R, K276R, K291R). In addition, p‐RK5‐HA‐Ub, pRK5‐HA‐Ubiquitin‐K48, and pRK5‐HA‐Ubiquitin‐K63 were purchased from Addgene (Watertown, MA, USA).

### Cell Lines and Cell Culture

Human GBM cell line (U87), other cancer cells (HCT116, PC3, MM.1s, SKOV3), and normal cells (HEK293T) were obtained from American Type Culture Collection (ATCC, Manassas, VA, USA). Human GBM cell lines (U251 and U343) were purchased from CLS Cell Lines Service GmbH (Eppelheim, Germany) and normal cell line (GM07492) was obtained from Coriell Institute for Medical Research (Camden, NJ, USA). All mouse embryonic fibroblast (MEF) cells were kindly provided by Tso‐Pang Yao (Duke University, Durham, NC, USA) and Joo‐Yong Lee (Chungnam National University, Daejeon, Korea).^[^ ^57^, ^58^ ^]^ Lentiviral system was used to establish knockdown and knockout stable cells. Lentiviruses were collected from HEK293T cells after they were transfected with lentiviral plasmids (short‐hairpin RNA (shRNA) or single guide RNA (sgRNA) containing plasmids) and packaging plasmids (pMD2G and psPAX2) using transfection reagent, lipofectamine 2000 (ThermoFisher, Waltham, MA, USA). Then, cells were infected with lentivirus, and infected cells were selected with 2 µg mL^−1^ puromycin (Enzo Life Sciences Inc., Farmingdale, NY, USA). pLKO‐based shRNA sets were purchased from Sigma‐Aldrich (St. Louis, MO, USA). The following shRNAs were used to establish knockdown cells. Human HDAC6 shRNA (#1: TRCN0000004839, #2: TRCN0000004842, #3: TRCN0000004843, #4: TRCN0000004840, #5: TRCN0000314976), human USP9X shRNA (#1: TRCN0000007361, #2: TRCN0000007362), human GS shRNA (TRCN0000343990), and for the non‐targeting control (NT), green fluorescent protein sequence was used. The knockout cells were established using lentiCRISPR v2 vector. The following sgRNAs were used to establish knockout cell lines. sgHDAC6: GGTGGAATCCTGGCCGGTTG, sgGS #1: AAATTCCACTCAGGCAACT‐CTGG, #2: TATTACTGTGGTGTGGGAGC, and for the non‐targeting (NT) control, green fluorescent protein sequence was used. sgUSP15: TGGTGATGCCCAGTCACTTA. Monoclonal HDAC6 knockout cells (sgHDAC6 #1, #2) were obtained after seeding and culturing single cells in each well of 96 well plates. The cells were cultured according to the instructions from ATCC in a humidified atmosphere of 95% air and 5% CO_2_ at 37 °C. DMEM and RPMI culture media and fetal bovine serum were purchased from Welgene Inc. (Gyeongsan, Korea). shRNA and sgRNA sequences are summarized in Table S3 (Supporting Information).

### Cell Viability Assay

Cell viability was examined with cell counting kit (CCK)‐8 assay (Dojindo Molecular Technologies, Inc., Kumamoto, Japan). 200 µL of cell suspension (3000 cells per well) was dispensed in 96‐well plates and reagents (HDAC inhibitor and USP9X inhibitor) were treated after overnight incubation with indicated concentrations for the indicated time. Then, 20 µL of WST‐8 reagent was added to each well and incubated for 3 h. Absorbance at 450 nm was measured using a microplate reader (TECAN, Mannedorf, Switzerland). Cell viability (%) was calculated by dividing with absorbance of the negative control. The calculated cell viability was analyzed statistically by Prism 8.0 (GraphPad Inc., San Diego, CA, USA). In addition, IC_50_ and GI_50_ were determined with nonlinear regression by Prism 8.0.

### Proliferation Assay

IncuCyte Live‐Cell Imaging System (Essen BioScience, Hertfordshire, UK) was used to monitor real‐time cell proliferation. 500 cells were seeded in each well of 96‐well plates. Phase‐contrast images were automatically obtained every 24 h, and monolayer confluence for individual images was acquired through a contrast‐based confluence algorithm.

### Clonogenic Assay

500 cells were seeded per well in a 6‐well plate and grown for 10–14 days. For glutamine starvation, the media was changed to glutamine free media (Welgene, LM001‐08) 3 days after seeding. When cells reached optimal confluence, colonies were visualized by staining with 0.5% crystal violet for 30 min and counted.

### Immunoblotting (IB) and Immunoprecipitation (IP)

For immunoblotting, each sample was separated by SDS‐PAGE. Proteins were transferred to nitrocellulose membranes using the wet transfer system. The membranes were incubated with corresponding primary antibodies at 4 °C overnight with constant agitation. Then, the membranes were incubated with HRP‐conjugated secondary antibodies (Jackson Immunoresearch, West Grove, PA, USA) for 3 h. The signals were visualized using enhanced chemiluminescence reagents (ThermoFisher, Waltham, MA, USA). For immunoprecipitation, a mixture composed of protein lysate (cells were incubated with 20 µm MG132 for 4 h before making cell lysate for ubiquitination assay), protein A/G bead in 50% slurry, and primary antibody was incubated at 4 °C overnight with constant agitation. Then, proteins were eluted from precipitated beads and subjected to immunoblotting. Protein levels were quantified by loading controls (α‐tubulin, GAPDH, or β‐actin), relative to the experimental control. If there were no experimental control, the protein amount in the first lane of each blot was used for normalization.

### RNA Isolation and Quantitative Real‐Time PCR (qRT‐PCR)

Total RNA was isolated using FavorPrep Total RNA Purification Mini Kit (Favorgen, Vienna, Austria) and reverse‐transcribed into cDNA. qRT‐PCR was performed with SYBR green using Applied Biosystems 7500 System (Applied Biosystems, Foster City, CA, USA). Relative mRNA expression levels were normalized to the mRNA level of GAPDH.

### The Primers Sequences Were as Follows

*HDAC6* forward: 5′‐ TTAGGCCTCCTGGA‐CATCAC ‐3′, *HDAC6* reverse: 5′‐ GCGGTGGATGGAGAAATAGA‐3′, *GLUL* forward: 5′‐TTCTGCTGGTGTAGCCAATC‐3′, *GLUL* reverse: 5′‐GGGCGACGATCTTCAAAGTAA‐3′, *NANOG* forward: 5′‐TCTTCCTGGTCCCCACAGTTT‐3′, *NANOG* reverse: 5′‐GCAAGAATAGTTCTCGGGATGAA‐3′, *SOX2* forward: 5′‐GCGGAGTGGAAACTTTT‐GTCC‐3′, *SOX2* reverse: 5′‐CGGGAAGCGTGTACTTATCCTT‐3′, *GAPDH* forward: 5′‐CATGAGAAGTATGACAACAGCCT‐3′, *GAPDH* reverse: 5′‐AGTCCTTCCACGATA‐CCAAAGT‐3′.

### Enzyme Activity Assay

GS activity was examined by colorimetric method using Glutamine Synthetase Activity Assay Kit (Biovision, #K2056), and HDAC6 activity was determined by fluorometric method using HDAC6 Activity Assay Kit (Biovision, #K466) according to the manufacturer's instruction.

### Immunofluorescence (IF)

2.0 × 10^5^ of U251 and U87 cells were seeded on the coverslip and fixed with 4% paraformaldehyde for 30 min. After fixation, cells were permeabilized with 0.2% triton‐X 100 for 25 min. Then, samples were blocked with 3% bovine serum albumin for 1 h at room temperature (RT), and the blocked samples were incubated with the indicated primary antibodies for 4 h at RT. The samples were incubated with Alexa Fluor 488‐ or Alexa Fluor 594 dye‐conjugated secondary antibodies (Jackson Immunoresearch, West Grove, PA, USA) in a blocking buffer for 2 h, and nuclei of cells were stained with DAPI for 5 min. The coverslips were mounted and analyzed with LSM710, a confocal laser scanning microscopy (ZEISS, Oberkochen, Germany).

### Apoptosis Assay

1.0 × 10^6^ of cells were seeded into 100 mm culture dishes and incubated with the indicated inhibitors (5 µm ACY738, 5 µm WP1130, and the combination of both inhibitors) for 24 h. After collecting and washing cells, apoptosis induced by the inhibitor treatment was examined with AnnexinV/Propidium Iodide (PI) staining using FITC Annexin V Apoptosis Detection Kit (BD Biosciences, 556547), according to the manufacturer's protocol. Then, stained cells were analyzed using flow cytometer (BD Biosciences, Franklin Lakes, NJ, USA).

### Metabolite Tracing and Extraction

1.5 × 10^6^ of established cells (U251 NT, shGS, shHDAC6, and shUSP9X) were seeded in 6‐well plates and incubated with glutamine‐free DMEM media (Welgene, LM001‐08) for 12 h. For ^13^C labeling, 2 mm of ^13^C_5_‐glutamine (Sigma, 605166) was supplemented. For ^15^N labeling, 0.8 mm ^15^N‐NH_4_Cl (Sigma, 299251) and 6 mm dimethyl‐ketoglutarate (Sigma, 349631) were supplemented. After 24 h incubation with isotopes, cells were harvested and metabolites were extracted with 500 µL of 80% ice‐cold methanol subjected to ultrasonic disruption for 30 s, with 5 s intervals between treatments. Then 400 µL of chloroform was added and shaken for 20 min. The samples were centrifuged at 14 000 × *g* for 20 min, and the upper phase was collected for freeze‐drying followed by re‐suspension with 100 µL of 0.2% formic acid in distilled water (DW).

### Liquid Chromatography and Mass Spectrometry (LC‐MS)

Metabolite analysis was conducted using a UHPLC‐MS/MS system combined with an LTQ Orbitrap Velos Pro (ThermoFisher, Waltham, MA, USA). A HILIC column (90 Å, 2.1 µm, 2.6 mm × 100 mm) from Thermo Fisher Scientific was used for separation. The mobile phase consisted of A) DW and B) 90% ACN each containing 10 mm NH_4_HCO_2_ and 0.2% formic acid. Gradient conditions were: 0.0–1.6 min = 30–100% B; 1.6–3.2 min = 100% B; 3.2–3.5 min = 100% to 30% B; 3.5–7 min = 30% B. The column oven was maintained at 30 °C, the injection volume was 10 µL, and the flow rate was 0.2 mL min^−1^. The mass spectrometer was operated in positive electrospray ionization mode for Gln, Glu, and IMP whereas negative electrospray ionization mode for α‐KG, and UMP. Selected reaction monitoring (SRM) was used for quantitative analytical assay while full‐scan MS2 was applied to confirm the fragment ions of selected precursor ions. The spray voltage was +3.9 kV and the collision energy was 30 eV. The ions used for the SRM analysis are shown in Table S2 (Supporting Information).

### Sphere Formation Assay

500 cells were seeded into ultra‐low attachment 6 well plate (Corning Life Sciences, Kennebunkport, ME, USA) and cultured with sphere culture media for 14 days. Sphere culture media was made of DMEM/F12 (1:1), 1% B27 supplement (Invitrogen, 1704044), 20 ng mL^−1^ of hEGF (Sigma, PHG0311), and 20 ng mL^−1^ of bFGF (Invitrogen, PHG0368). The spheres (>100 µm in diameter) in each well were counted.

### Animal Experiments

All animal experimental protocols were approved by the Animal Institutional Animal Care and Use Committee of Yonsei University (No. IACUC‐A‐202211‐1561‐01 and IACUC‐A‐202409‐1919‐01). 4 weeks old female Balb/c nude mice were obtained from Orient Bio (Seongnam, Korea) and maintained under a specific pathogen‐free facility of Yonsei University (Seoul, Korea). Mice were bred at constant humidity and RT (24 °C) and at a 12 h light/dark cycle. Mice were allowed to sterilized rodent chow and water ad libitum. For the subcutaneous xenograft model, randomized mice (*n* = 6 per group) were subcutaneously injected into the flanks with 2.5 × 10^6^ of U251 cells (NT, shHDAC6 #1, #2, and shUSP9X #1, #2), which were suspended in 100 µL of PBS containing 50% Matrigel (Corning Life Sciences, Kennebunkport, ME, USA). Tumor sizes were estimated every 3–4 days after tumors became palpable. When the tumor volume reached 1000 mm^3^, mice were sacrificed in a 7.5% CO_2_ chamber, and tumor tissues were isolated and subjected to immunohistochemistry and other experiments. For the intracranial xenograft model, randomized mice (*n* = 5 per group) were injected in the subcortical region (ML: 2 mm, AP: 1 mm, DV: 3 mm) with 3 × 10^5^ of luciferase‐overexpressing U251 cells (NT, shHDAC6 #1, #2, and shUSP9X #1, #2), suspended in 4 µL of PBS, using a Hamilton syringe. Bioluminescence images were obtained weekly for 3 weeks after cell implantation using in vivo imaging system, IVIS‐Lumina XRMS Series III (Perkin Elmer, Waltham, MA, USA). After 3 weeks after cell implantation, tumor tissues were isolated and subjected to H&E and immunohistochemistry staining.

### H&E Staining and Immunohistochemistry (IHC)

Following sacrificing mice, tumor tissues were harvested and fixed with 4% paraformaldehyde. Tumor tissues were embedded in paraffin and sectioned. For H&E staining, tissue sections were stained with Mayer's hematoxylin (Dako, S3309), followed by staining with eosin (Sigma, 318906). For IHC staining, tissue sections were incubated with anti‐GS (Genetex, GTX109121) and Ki‐67 (Santacruz, sc‐23900) antibodies at 4 °C overnight. To visualize the staining, ABC‐HRP kit (Vector Laboratories, PK‐7200) and DAB substrate kit (Vector Laboratories, SK‐4100) were used. For counter staining, Mayer's hematoxylin was used. H&E and IHC stained sections were mounted with mounting medium (Biognost, BMT‐100) using cover, and bright‐field images were obtained with a microscope digital camera, DP73 (Olympus, Tokyo, Japan) and microscope slide scanner, Ocus40 (Grandium, Tampere, Finland).

### Tissue Microarray (TMA)

Brain GBM clinical TMAs (GL806‐L64 and GL805‐L51) were purchased from Tissue Array.com (Derwood, MD, USA) and were subjected to IHC. Antibodies against HDAC6 (Bethyl, A301‐342A), USP9X (Santa Cruz Biotechnology, sc‐365353), and GS (Genetex, GTX109121) were used. To visualize the stained slides, pannoramic digital slide scanner (Pannoramic MIDI) was used (3DHISTECH, Budapest, Hungary).

### Measurement of Glutamine and Glutamate from GBM Xenograft Tissue

10 mg of tumor tissues from U251‐derived subcutaneous xenograft were homogenized with 100 µL of the enclosed lysis buffer in kit and glutamine and glutamate levels were determined with the colorimetric method using Glutamine Assay Kit (ab197011) and Glutamate Assay Kit (ab83389) according to the manufacturer's instruction.

### Statistical Analysis

Statistical significance was determined using GraphPad Prism software (version 8.0, GraphPad Software). The difference between two groups was assessed by student's *t*‐test (two‐tailed), whereas one‐way analysis of variance (ANOVA) was used for comparisons among multiple groups. For analyses involving two independent variables, such as those in CHX assay quantification and tumor volume graphs, a two‐way ANOVA was employed. Pearson's correlation analysis was used to evaluate correlations, and Kaplan–Meier analyses were evaluated with the log‐rank test. The graphs in this study were presented with the mean ± standard deviation (SD) of more than three independent experiments. Sample sizes (*n*) for each statistical analysis were provided in the figure legends and *p* values (*p*) were indicated in the figures.

## Conflict of Interest

The authors declare no conflict of interest.

## Author Contributions

G.W.K.: Conceptualization, methodology, formal analysis, data curation, visualization, funding acquisition, writing‐original draft, writing‐review, and editing. M.C.: Formal analysis. H.T.M.O.: Formal analysis. J.Y.: Formal analysis, writing‐review, and editing. Y.H.J.: Formal analysis, writing‐review, and editing. S.W.L.: Writing‐review and editing. S.Y.O.: Writing‐review and editing. M‐J.K.: Methodology and formal analysis. Y.K.: Methodology and formal analysis. S.H.K.: Conceptualization, supervision, project administration, funding acquisition, writing‐review, and editing.

## Supporting information



Supporting Information

## Data Availability

The data that support the findings of this study are available from the corresponding author upon reasonable request.
