# Supplementary material for: HDAC6 and USP9X Control Glutamine Metabolism by Stabilizing GS to Promote Glioblastoma Tumorigenesis
Source: Adv Sci (Weinh). 2025 Mar 31;12(25):2501553. doi: 10.1002/advs.202501553 (PMC12224959; doi:10.1002/advs.202501553)
Supplement: Supplementary file 1 — Supporting Information [file ADVS-12-2501553-s001.docx]

**Supporting Information**

**HDAC6 and USP9X Control Glutamine Metabolism by Stabilizing GS to Promote Glioblastoma Tumorigenesis**

*Go Woon Kim^1^, Minhae Cha^1^, Hien Thi My Ong^2,3^, Jung Yoo^1^, Yu Hyun Jeon^1^, Sang Wu Lee^1^, Soo Yeon Oh^1^, Min-Jung Kang^2,3^, Youngsoo Kim^1^, and So Hee Kwon^1*^*

**Figure S1.** Upregulation of GS in GBM is associated with poor prognosis. A) Representative images and the number of stained samples from IHC staining of GS in glioma and normal tissues. Data were obtained from the Human Protein Atlas database. B) Percentage of patients with moderate to strong staining of GS in various cancers. Data were obtained from the Human Protein Atlas. C) GEO profile analysis (GSE15824) showing the GLUL gene expression in brain cancers, including GBM. D, E) Kaplan-Meier survival analysis of primary GBM samples from the GEO dataset (GSE42669), stratified by GLUL expression for overall survival (OS) and progression-free survival (PFS). Data are shown as mean ± SD.


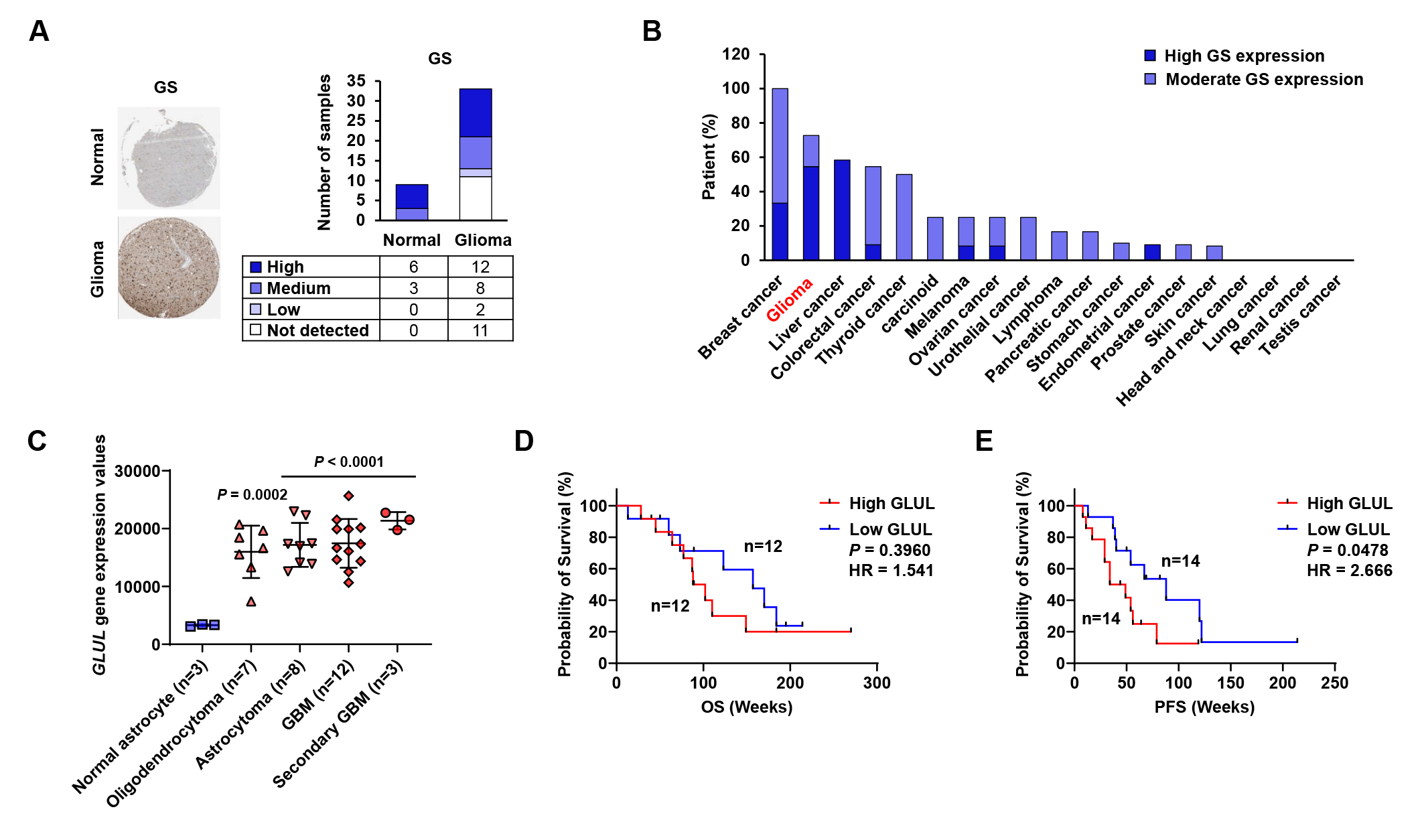

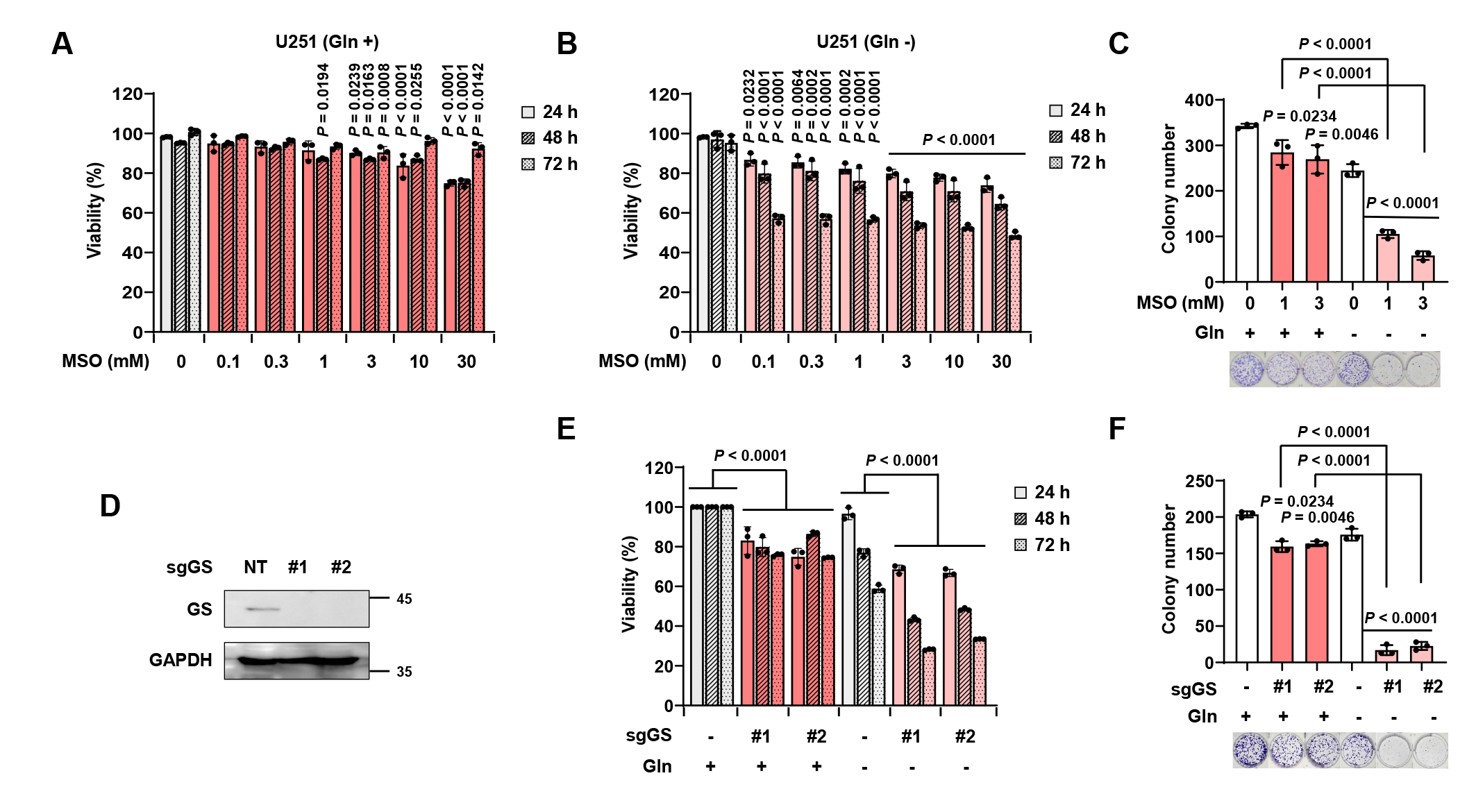


**Figure S2.** Targeting GS suppresses GBM cell growth in vitro. A, B) Cell viability of U251 cells treated with methionine sulfoximine (MSO), a GS inhibitor, under conditions of glutamine repletion (A) and depletion (B). C) Colony formation analysis of MSO-treated U251 cells incubated in Gln+/- media. D) IB analysis showing the GS protein expression in GS knockout U251 cells. E, F) Cell viability (E) and colony formation analysis (F) of GS knockout U251 cells incubated in Gln+/- media. Data are shown as mean ± SD. *n*=3.

**
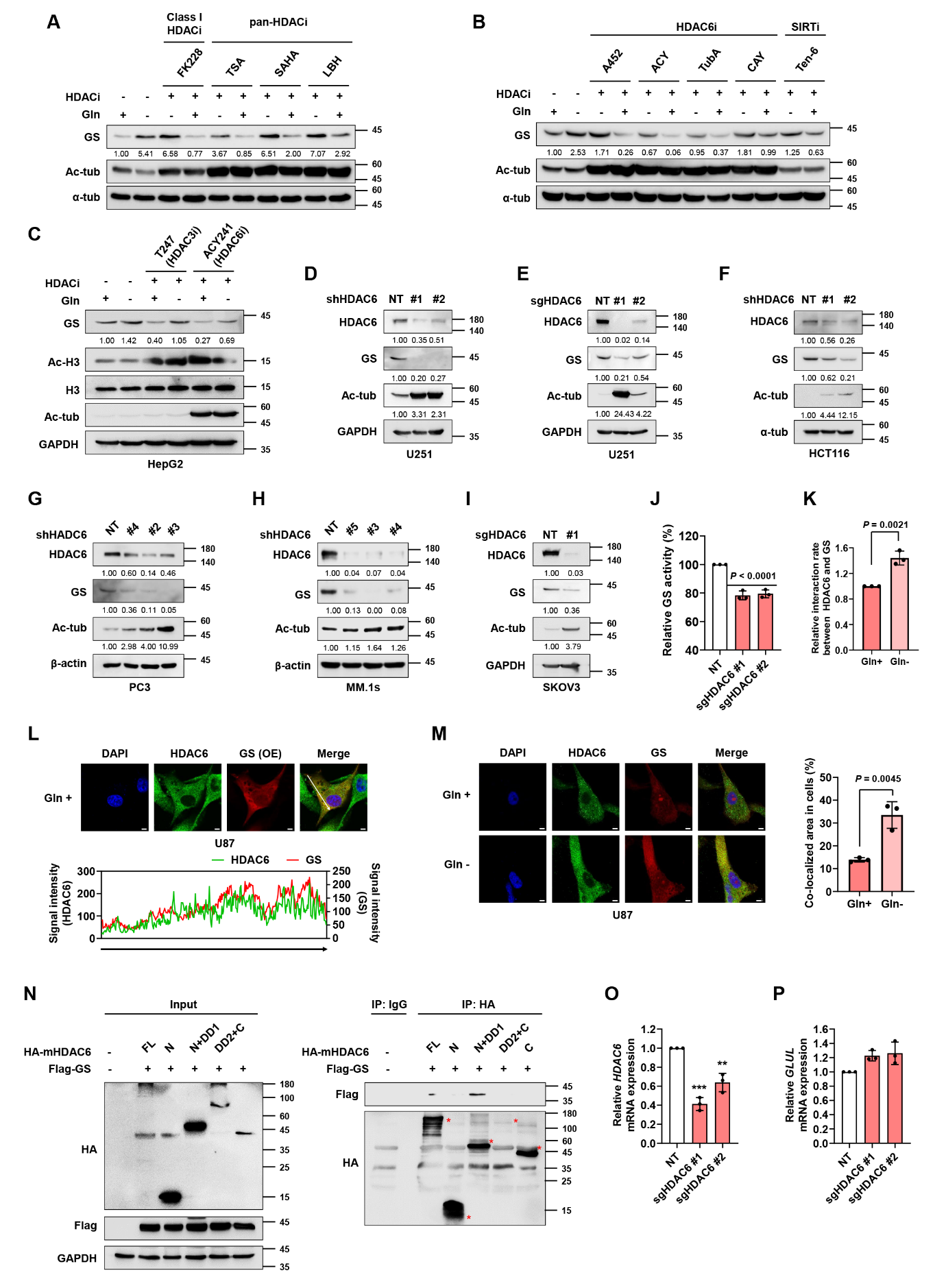
**

**Figure S3.** HDAC6 stabilizes GS by directly interacting with GS. A) HDAC inhibitor screening using 0.5 μM FK228 and 2 μM pan-HDAC inhibitors including Trichostatin A (TSA), SAHA, and LBH589 (LBH), in U251 cells. Cells were incubated with the indicated HDAC inhibitors for 24 h. B) HDAC inhibitor screening using 10 μM Tenovin-6 (Ten-6), and 4 μM HDAC6-selective inhibitors including A452, ACY241 (ACY), Tubastatin A (TubA), and CAY10603 (CAY) in U251 cells. Cells were incubated with the indicated concentration of HDAC inhibitors for 24 h. C) HDAC inhibitor screening with 2 μM T247 and 2 μM ACY241 in HepG2 cells. Cells were incubated with the indicated HDAC inhibitors for 24 h. D) IB analysis showing the GS protein level in HDAC6 knockdown U251 cells. E) IB analysis showing the GS protein levels in HDAC6 knockout U251 cells. F) IB analysis showing the GS protein levels in HDAC6 knockdown HCT116, a colorectal cancer cell. G) IB analysis showing the GS protein level in HDAC6 knockdown PC3, a prostate cancer cell. H) IB analysis showing the GS protein level in HDAC6 knockdown MM.1s, a multiple myeloma cell. I) IB analysis showing the GS protein level in HDAC6 knockout SKOV3, an ovarian cancer cell. J) GS activity in HDAC6 knockout U251 cells. GS activity was calculated relative to the control group. K) Quantitative graph showing interaction rate between HDAC6 and GS, related to Figure 1G. L) Representative immunofluorescence images and signal intensity profiles across the line in the merged image showing co-localization of HDAC6 and ectopically expressed GS in U87 cells. Scale bar, 5 μm. (M) Representative immunofluorescence images and quantitative graph showing co-localization of HDAC6 and GS in glutamine-starved U87 cells incubated in Gln +/- media for 48 h. Scale bar, 5 μm. N) IP analysis showing the interaction domain of HDAC6 binding to GS in HEK293T cells. Red-colored asterisks indicate immunoprecipitated protein bands. O, P) mRNA levels of *HDAC6* (O) and *GLUL* (P) analyzed with qRT-PCR in HDAC6 knockout U251 cells. Data are shown as mean ± SD. *n*=3.

**
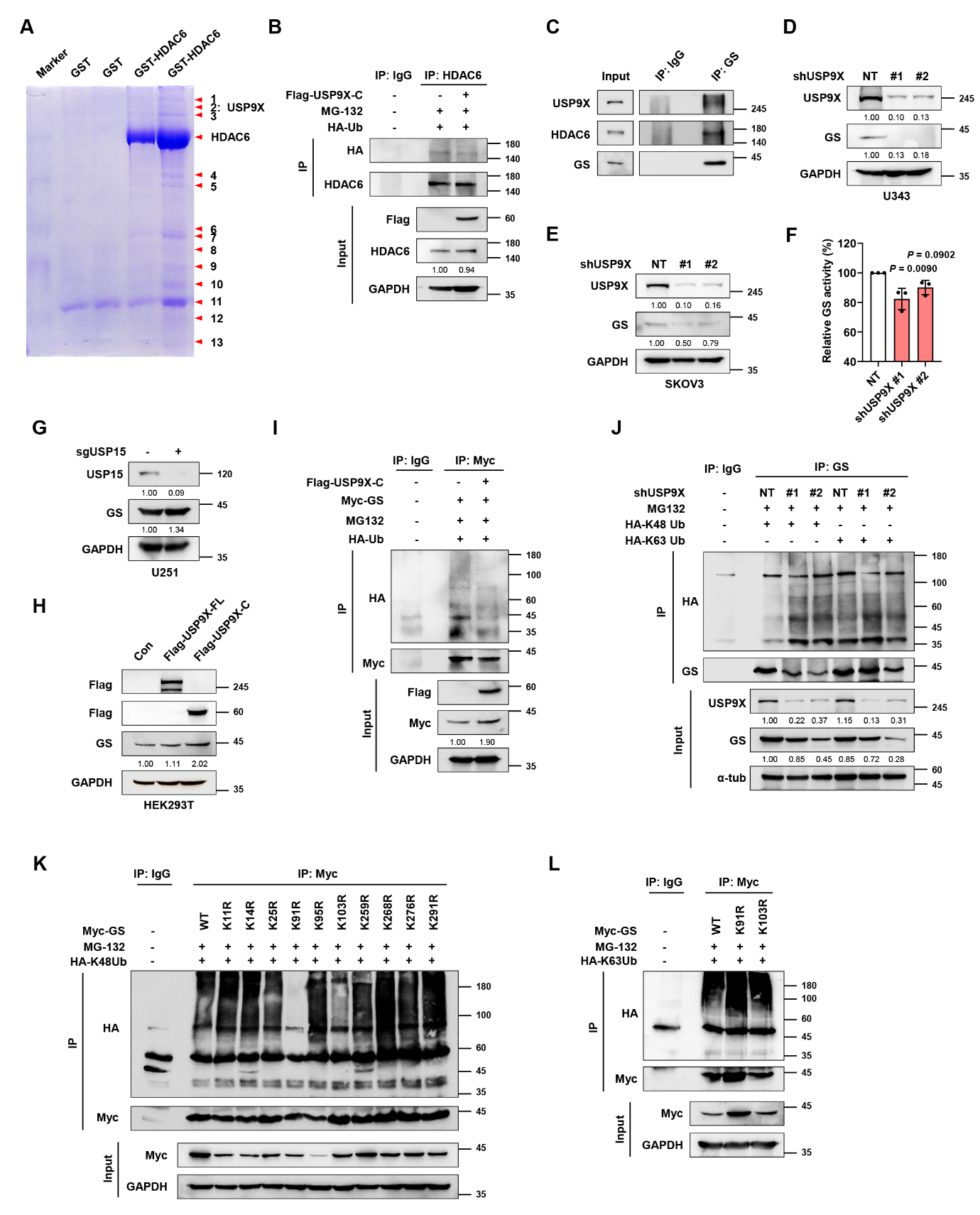
**

**Figure S4.** USP9X stabilizes GS protein via deubiquitination. A) Coomassie blue staining showing the purification of GST-tagged HDAC6 protein in HeLa cells. B) Ubiquitination assay showing the changes in HDAC6 ubiquitination in USP9X-peptidase domain containing c-term (USP9X-C)-overexpressing U251 cells. C) IP analysis showing the interaction of USP9X, HDAC6, and GS in U251 cells. D) IB analysis showing the GS protein levels in USP9X knockdown U343, a GBM cell. E) IB analysis showing the GS protein levels in USP9X knockdown SKOV3, an ovarian cancer cell. F) GS activity in USP9X knockdown U251 cells. GS activity was calculated relative to the control group. G) IB analysis showing the GS protein levels in USP15 knockout U251 cell. H) IB analysis showing the GS protein level in USP9X-Full length (FL) or USP9X-C overexpressed HEK293T cells. I) Ubiquitination assay showing the ubiquitination of GS in USP9X-C-overexpressing U251 cells. J) Ubiquitination assay showing the changes in GS ubiquitination in USP9X knockdown U251 cells. Cells were transfected with HA-tagged K48- or K63-linked ubiquitin. K) Ubiquitination assay showing K48-linked ubiquitination of GS mutants in U251 cells. L) Ubiquitination assay showing the change of K63-linked ubiquitination of GS mutants in U251 cells. Data are shown as mean ± SD. *n*=3.


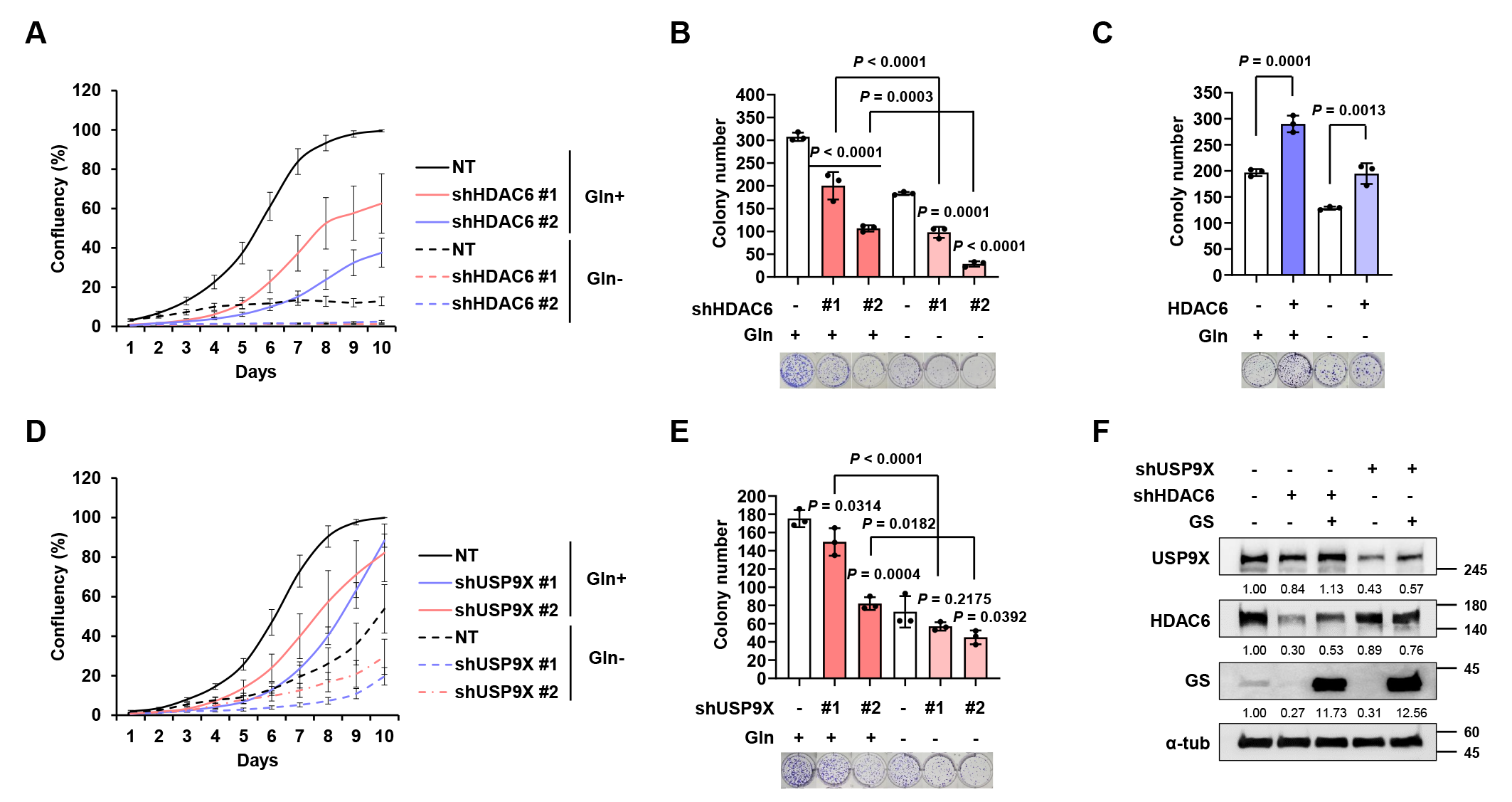


**Figure S5.** Targeting HDAC6 and USP9X suppresses GBM cell growth. A) Cell proliferation analysis of HDAC6 knockdown U251 cells incubated in Gln+ (represented by solid lines)/Gln- (represented by dotted lines) media (*n*=5). B) Colony formation analysis of HDAC6 knockdown U251 cells incubated in Gln+/- media. C) Colony formation analysis of HDAC6-overexpressing U251 cells incubated in Gln+/- media supplemented with 4 mM glutamate and 0.8 mM NH_4_Cl. D) Cell proliferation of USP9X knockdown U251 cells incubated in Gln+ (represented by solid lines)/Gln- (represented by dotted lines) media (n=5). E) Colony formation analysis of USP9X knockdown U251 cells incubated in Gln+/- media. F) IB analysis showing GS introduction into the HDAC6 and USP9X knockdown U251 cells. Data are shown as mean ± SD. *n*=3, unless otherwise noted.


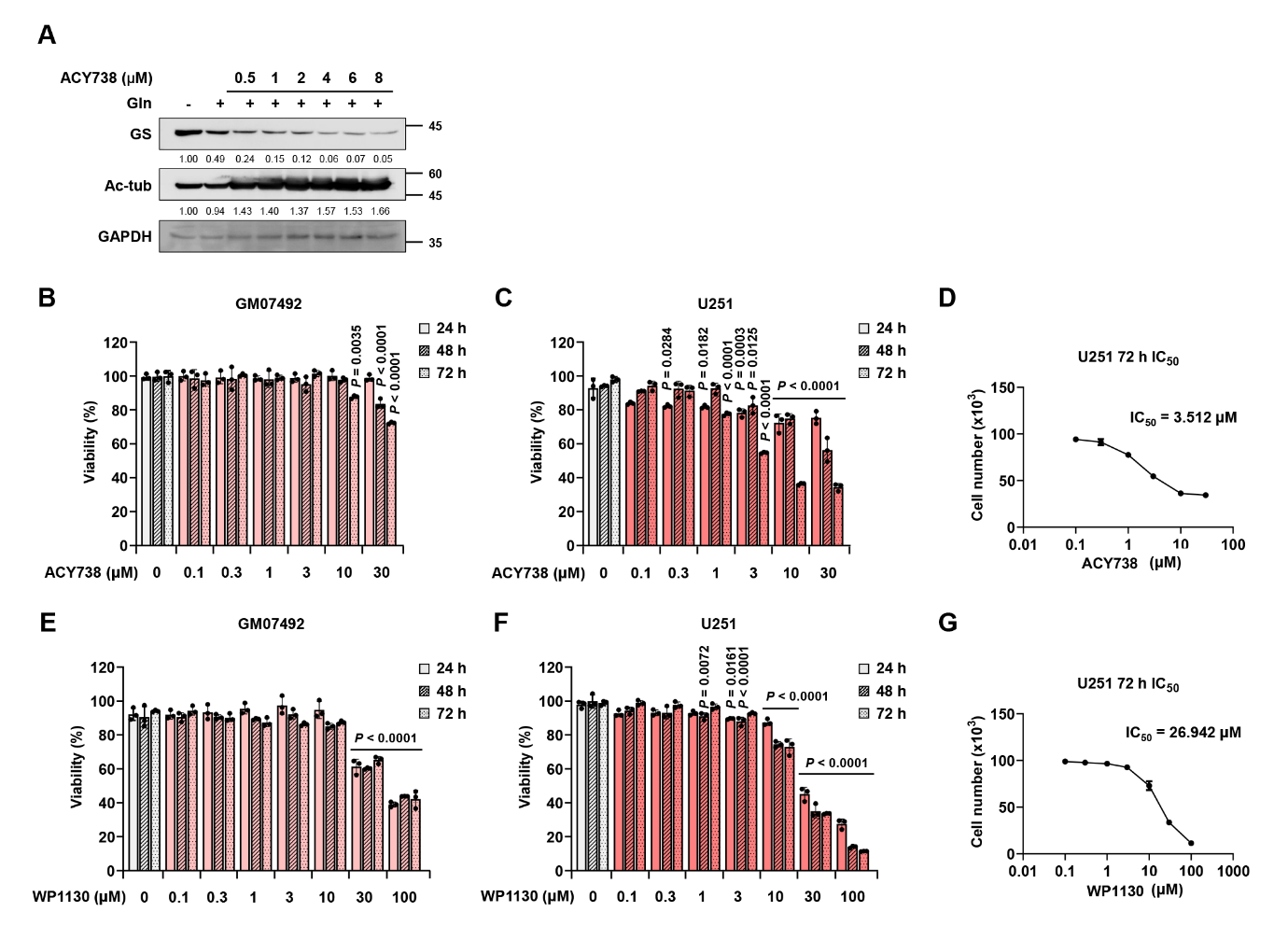


**Figure S6.** Pharmacological inhibition of HDAC6 and USP9X suppresses GBM cell growth. A) IB analysis showing the effects of ACY738 treatment on GS expression in U251 cells. B, C) Cell viability analysis of ACY738-treated normal cell line GM07492 (B) and GBM cell line U251 (C) incubated in Gln+/- media. D) IC_50_ of ACY738 after 72 h of incubation in U251 cells. E, F) Cell viability analysis of WP1130-treated GM07492 (E) and U251 (F) incubated in Gln+/- media. G) IC_50_ of WP1130 after 72 h of incubation in U251 cells. Data are shown as mean ± SD. *n*=3.


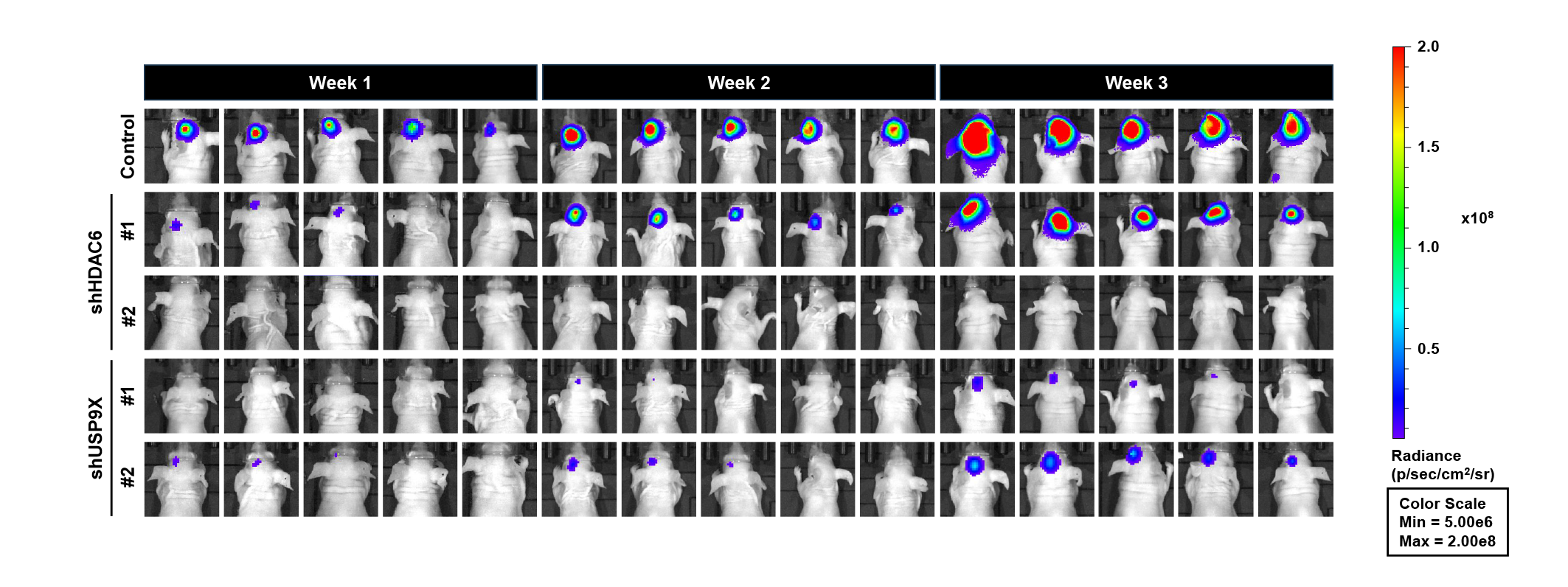


**Figure S7.** Silencing of HDAC6 and USP9X reduces intracranial GBM tumors. Bioluminescence images of U251-derived intracranial xenograft mice taken at weeks 1, 2, and 3 after cell implantation (n=5).


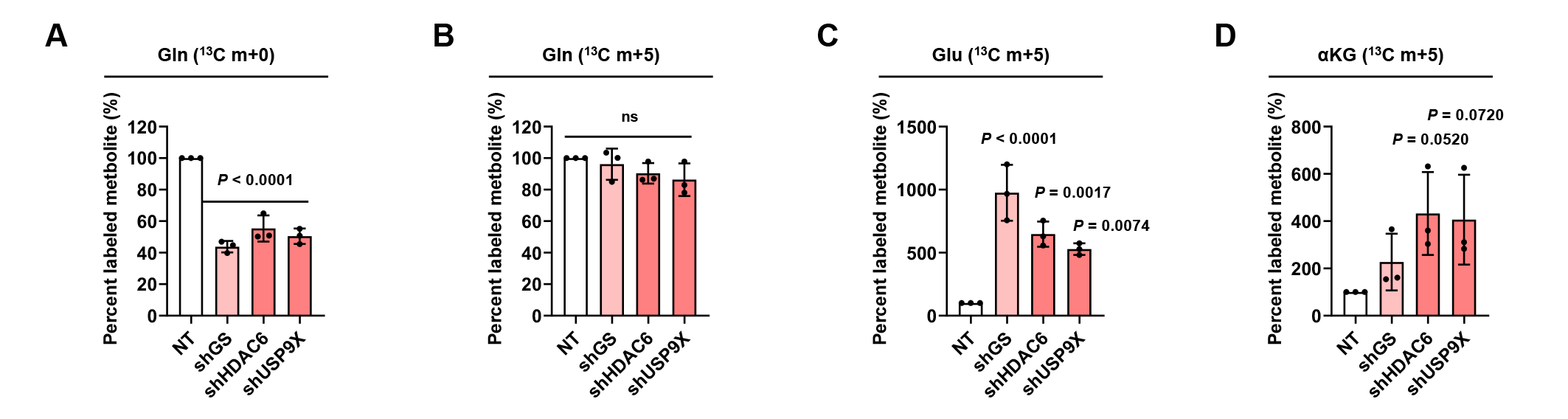


**Figure S8**. Ablation of USP9X and HDAC6 impairs de novo glutamine synthesis. A-D) Relative metabolite levels of non-labeled Gln, ^13^C_5_-glutamine-derived m+5 Gln, m+5 Glu, and m+5 αKG normalized to NT control cells. Cells were glutamine-depleted for 12 h before labeling and incubated in glutamine-free media containing 2 mM ^13^C_5_-glutamine for 24 h. Data are shown as mean ± SD. ns, not significant; *n*=3.


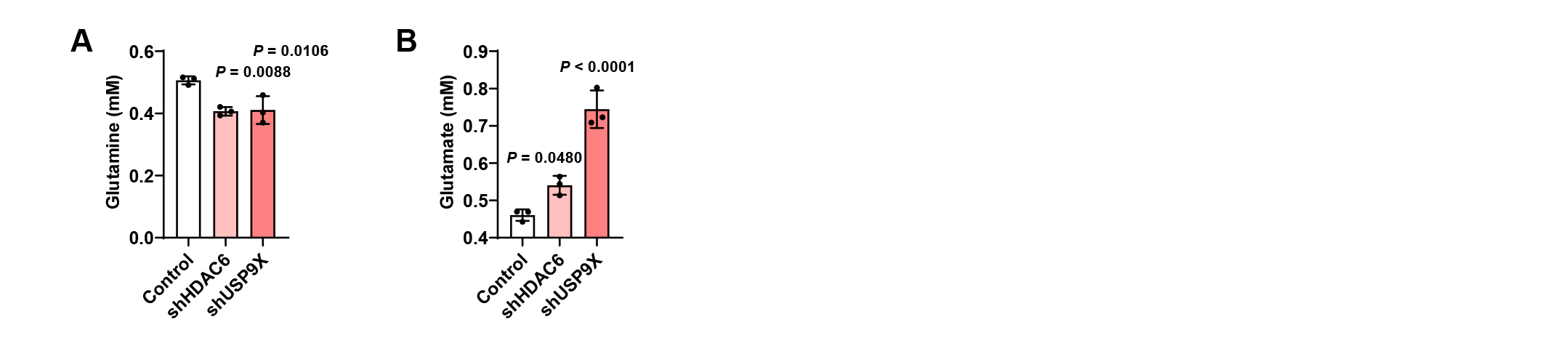


**Figure S9.** Glutamine and glutamate metabolite levels are reduced in HDAC6 and USP9X knockdown U251-derived xenograft GBM tissue. A, B) Relative glutamine (A) and glutamate (B) levels in HDAC6 and USP9X knockdown U251-derived xenograft GBM tissues. Data are shown as mean ± SD. *n*=3.

**Figure S10.** Silencing of HDAC6 and USP9X diminishes stem cell-like properties of GBM cells. A) Volcano plot showing the significantly upregulated and downregulated genes in GBM stem-like cells compared to conventional glioma cell lines (fold change > 1.5 and *P*-value < 0.01). Data were obtained from GSE23806. B-D) GEO profile analysis (GSE23806) showing upregulation of *GLUL* (B), *HDAC6* (C), and *USP9X* (D) in stem-like GBM samples, including GBM stem-like cells, GBM stem-like neurospheres, and primary tumors compared to conventional glioma cell lines. E, F) GEO profile analysis (GSE23806) showing the positive correlation between expression of *GLUL*-*HDAC6* (E) and *GLUL*-*USP9X* (F) in GBM samples (*n*=61). G, H) Representative images (G) of spheres derived from control, HDAC6 knockdown, and USP9X knockdown U251 cells. Scale bar, 100 μm. Spheres were quantified after 7 days of incubation (H) (*n*=3). I, J) mRNA levels of *Nanog* (I) and *Sox2* (J), which are stemness markers, upon knockdown of HDAC6 and USP9X in U251 cells (*n*=3). K) IB analysis showing protein levels of stemness markers in HDAC6 and USP9X knockdown U251 cells. Data are shown as mean ± SD.


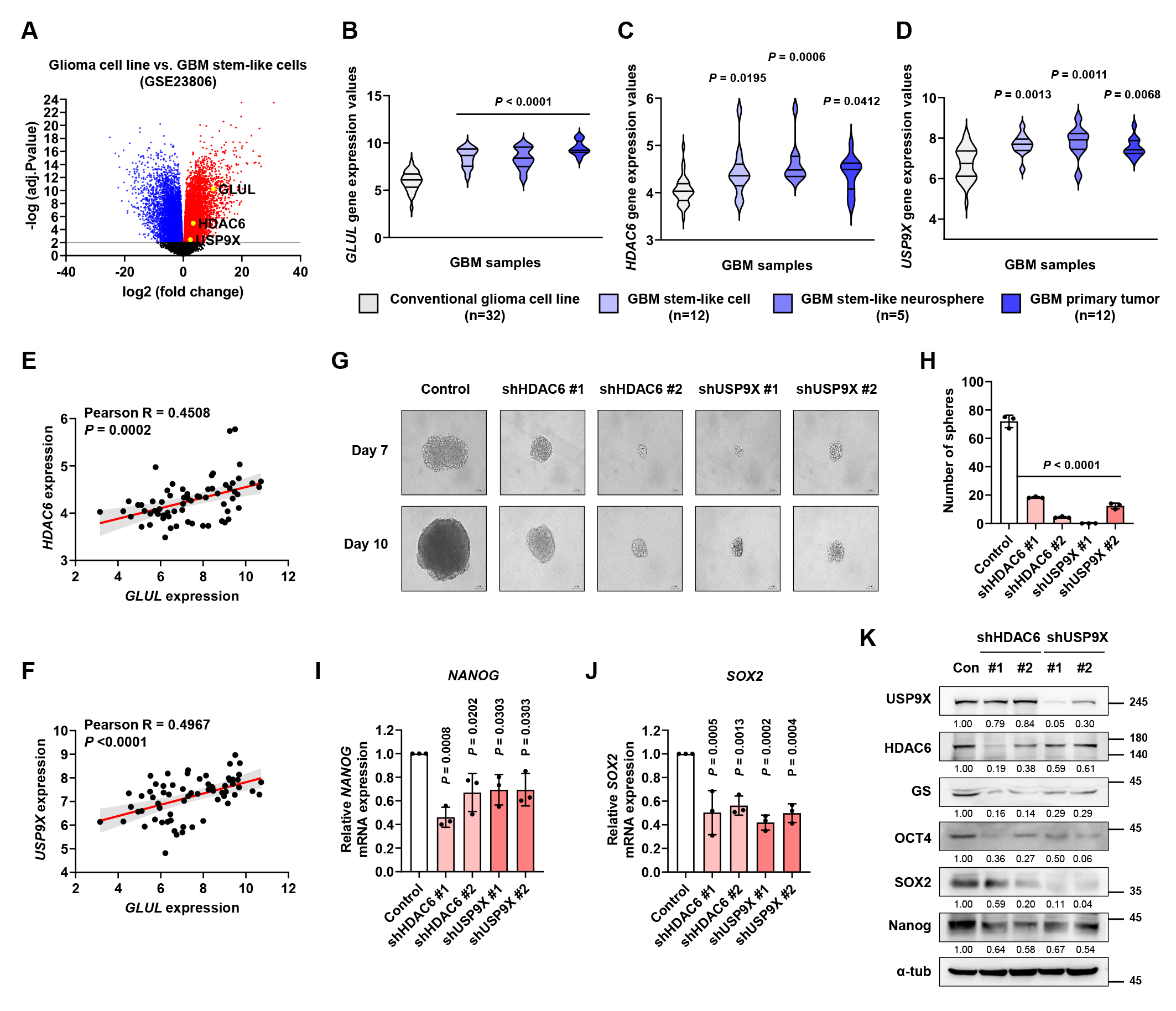


**-**


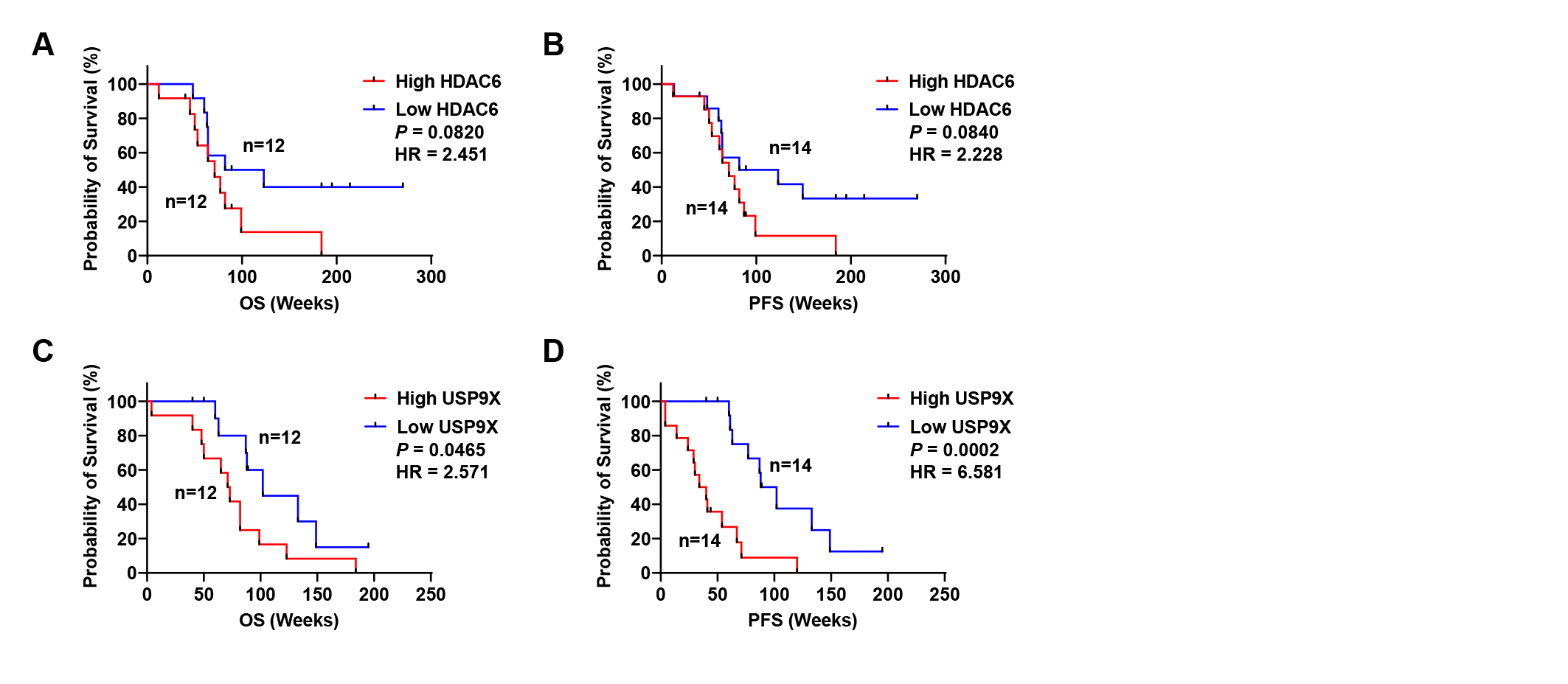


**Figure S11.** Survival analysis of GBM patients with high HDAC6 or USP9X expression. A, B) Kaplan-Meier survival analysis of primary GBM samples from the GEO dataset (GSE42669) stratified by HDAC6 expression for overall survival (OS) and progression-free survival (PFS). C, D) Kaplan-Meier survival analysis of primary GBM samples from the GEO dataset (GSE42669) stratified by USP9X expression for overall survival (OS) and progression-free survival (PFS).

**Table S1.** List of HDAC6-interacting proteins analyzed by IP-MS

| **Spot** | **Protein identified** | **Access #** | **Description** | **MW** |
| --- | --- | --- | --- | --- |
| 1 | dyhc_human | (Q14204) | Dynein heavy chain, cytosolic (DYHC) - 527286.0 | 527.0 |
| 1 | prkd_human | (P78527) | DNA-dependent protein kinase catalytic subunit - 469140.9 | 469.0 |
| 1 | q9ueg1 | (Q9UEG1) | Polyubiquitin- 68497.5 | 68.5 |
| 2 | fafx_human | (Q93008) | Probable ubiquitin carboxyl-terminal hydrolase FAF-X (USP9X) - 289622.7 | 289.6 |
| 3 | myha_human | (P35580) | Myosin heavy chain, nonmuscle type B -228939.3 | 229.0 |
| 3 | myh9_human | (P35579) | Myosin heavy chain, nonmuscle type A - 226532.2 | 226.5 |
| 3 | ubiq_human | (P02248) | Ubiquitin - 8567 | 8.5 |
| 4 | q9udx0 | (Q9UDX0) | Oxoglutarate dehydrogenase (lipoamide) -115935.3 | 115.9 |
| 4 | my1d_human | (O94832) | Myosin Id - 116202.2 | 116.2 |
| 4 | p137_human | (Q14444) | GPI-anchored protein p137 (p137GPI) - 72751.7 | 72.8 |
| 5 | sfpq_human | (P23246) | Splicing factor, proline-and glutamine-rich - 76149.3 | 76.1 |
| 5 | nucl_human | (P19338) | Nucleolin (Protein C23) - 76212.9 | 76.2 |
| 5 | odo1_human | (Q02218) | 2-oxoglutarate dehydrogenase E1 component, mitochondrial -113475.5 | 113.5 |
| 5 | q8n922 | (Q8N922) | Hypothetical protein FLJ38508 (Fragment) - 82741.4 | 82.7 |
| 5 | ef2_human | (P13639) | Elongation factor 2 (EF-2) - 95207.0 | 95.2 |
| 6 | hs7c_human | (P11142) | Heat shock cognate 71 kDa protein -70898.1 | 70.9 |
| 6 | odp2_human | (P10515) | Dihydrolipoamide acetyltransferase component of py - 65781.0 | 65.8 |
| 6 | hs71_human | (P08107) | Heat shock 70 kDa protein 1 (HSP70.1) - 70052.4 | 70.1 |
| 6 | pab1_human | (P11940) | Polyadenylate-binding protein 1 - 70670.10 | 70.7 |
| 7 | hs71_human | (P08107) | Heat shock 70 kDa protein 1 (HSP70.1) - 70052.2 | 70.1 |
| 7 | hs7h_human | (P34931) | Heat shock 70 kDa protein 1-HOM (HSP70-HOM) - 70052.2 | 70.1 |
| 7 | q86yi5 | (Q86YI5) | Similar to dihydrolipoamide S-acetyltransferase precursor - 68996.7 | 69.0 |
| 7 | hs72_human | (P54652) | Heat shock-related 70 kDa protein 2 (Heat shock 70) - 70021.0 | 70.0 |
| 7 | dd17_human | (Q92841) | Probable RNA-dependent helicase p72 -72371.4 | 72.3 |
| 8 | hs71_human | (P08107) | Heat shock 70 kDa protein 1 (HSP70.1) - 70052.2 | 70.1 |
| 8 | g3bp_human | (Q13283) | Ras-GTPase-activating protein binding protein 1 - 52164.4 | 52.2 |
| 8 | o88477 | (O88477) | Coding region determinant binding protein - 63450.6 | 63.5 |
| 8 | odp2_human | (P10515) | Dihydrolipoamide acetyltransferase component of pyruvate dehydrogenase complex, mitochondrial - 65781.0 | 65.8 |
| 8 | q96a50 | (Q96A50) | Hypothetical protein - 66276.4 | 66.3 |
| 9 | r54_human | (Q15233) | 54 kDa nuclear RNA- and DNA-binding protein - 54100.4 | 54.1 |
| 9 | dldh_human | (P09622) | Dihydrolipoamide dehydrogenase, mitochondrial - 54150.2 | 54.1 |
| 9 | tba1_human | (P05209) | Tubulin alpha-1 chain (Alpha-tubulin 1) - 50151.6 | 50.2 |
| 9 | vime_human | (P08670) | Vimentin - 53554.5 | 53.6 |
| 9 | sera_human | (O43175) | D-3-phosphoglycerate dehydrogenase (EC 1.1.1.95) - 56650.7 | 56.7 |
| 9 | q9y3y6 | (Q9Y3Y6) | Hypothetical protein (Fragment) - 33135.9 | 33.1 |
| 10 | odo2_human | (P36957) | Dihydrolipoamide succinyltransferase component of 2-oxoglutarate dehydrogenase complex, mitochondrial - 48640.4 | 48.6 |
| 10 | tbb5_human | (P05218) | Tubulin beta-5 chain - 49670.8 | 49.7 |
| 10 | q86tq8 | (Q86TQ8) | Human full-length cDNA 5-PRIME end of clone CS0DB006YE12 - 32807.9 | 32.8 |
| 10 | tbb2_human | (P05217) | Tubulin beta-2 chain - 49831.0 | 49.8 |
| 10 | odpx_human | (O00330) | Pyruvate dehydrogenase protein X component, mitochondrial - 54122.3 | 54.1 |
| 10 | tba1_human | (P05209) | Tubulin alpha-1 chain (Alpha-tubulin 1) - 50151.6 | 50.1 |
| 10 | odb2_human | (P11182) | Lipoamide acyltransferase component of branched-chain alpha-keto acid dehydrogenase complex, mitochondrial - 53487.1 | 53.5 |
| 10 | dldh_human | (P09622) | Dihydrolipoamide dehydrogenase, mitochondrial - 54150.2 | 54.2 |
| 10 | ef1g_human | (P26641) | Elongation factor 1-gamma (EF-1-gamma) - 50118.8 | 50.1 |
| 10 | atpa_human | (P25705) | ATP synthase alpha chain, mitochondrial precursor - 59750.6 | 59.8 |
| 10 | roh1_human | (P31943) | Heterogeneous nuclear ribonucleoprotein H - 49229.5 | 49.3 |
| 11 | ef1g_human | (P26641) | Elongation factor 1-gamma (EF-1-gamma) - 50118.10 | 50.1 |
| 11 | ef11_human | (P04720) | Elongation factor 1-alpha 1 (EF-1-alpha-1) - 50140.11 | 50.1 |
| 11 | odo2_human | (P36957) | Dihydrolipoamide succinyltransferase component of 2-oxoglutarate dehydrogenase complex, mitochondrial - 48640.4 | 48.6 |
| 11 | yb1_human | (P16991) | Nuclease sensitive element binding protein - 35926 | 35.9 |
| 11 | dja1_human | (P31689) | DnaJ homolog subfamily A member 1 (Heat shock 40 k) - 44868.4 | 44.9 |
| 12 | ef1g_human | (P26641) | Elongation factor 1-gamma (EF-1-gamma) - 50118.10 | 50.1 |
| 12 | odpa_human | (P08559) | Pyruvate dehydrogenase E1 component alpha subunit - 43295.6 | 43.3 |
| 12 | ef11_human | (P04720) | Elongation factor 1-alpha 1 (EF-1-alpha-1) - 50140.11 | 50.1 |
| 12 | actb_human | (P02570) | Actin, cytoplasmic 1 (Beta-actin) - 41736.7 | 41.7 |
| 12 | q9bwd4 | (Q9BWD4) | Interleukin enhancer binding factor 2, 45kD - 43062.1 | 43.1 |
| 13 | odpb_human | (P11177) | Pyruvate dehydrogenase E1 component beta subunit - 39219.6 | 39.2 |
| 13 | o43813 | (O43813) | SEVENTRANSMEMBRANE-domain protein (LANC-like protein 1) - 45283.1 | 45.3 |
| 13 | q96fj5 | (Q96FJ5) | Similar to RIKEN cDNA 2810403L02 gene (Fragment) - 39444.0 | 39.4 |
| 13 | odpa_human | (P08559) | Pyruvate dehydrogenase E1 component alpha subunit - 43295.6 | 43.3 |
| 13 | roa2_human | (P22626) | Heterogeneous nuclear ribonucleoproteins A2/B1 - 37429.9 | 37.4 |
| 13 | ef1g_human | (P26641) | Elongation factor 1-gamma (EF-1-gamma) - 50118.8 | 50.1 |
| 13 | ef11_human | (P04720) | Elongation factor 1-alpha 1 (EF-1-alpha-1) - 50140.11 | 50.1 |
| 13 | o14979 | (O14979) | JKTBP2 (hnRNP JKTBP) (A+U-rich element RNA binding factor) - 46437.5 | 43.5 |
| 13 | rl6_human | (Q02878) | 60S ribosomal protein L6 (TAX-responsive enhancer - 32596.7 | 32.6 |
| 13 | dhc3_human | (O75828) | Carbonyl reductase [NADPH] 3 (EC 1.1.1.184) (NADPH) - 30719.1 | 30.7 |
| 13 | mlrm_human | (P19105) | Myosin regulatory light chain 2, nonsarcomeric - 19662.9 | 19.7 |
| 13 | rs18_human | (P25232) | 40S ribosomal protein S18 (KE-3) - 17718.7 | 17.7 |
| 13 | calm_human | (P02593) | Calmodulin - 16706.4 | 16.7 |
| 13 | rs24_human | (P16632) | 40S ribosomal protein S24 (S19) - 15423.2 | 15.4 |
| 13 | cof1_human | (P23528) | Cofilin, non-muscle isoform (18 kDa phosphoprotein) - 18502.5 | 18.5 |
| 13 | rs10_human | (P46783) | 40S ribosomal protein S10 - 18897.8 | 18.9 |
| 13 | rl35_human | (P42766) | 60S ribosomal protein L35 - 14420.3 | 14.4 |

**Table S2.** The MRM transitions and optimized analytical conditions

| **Metabolites** | **Scan mode** | **Precursor ions** | **Fragment ions** | **Ion transition of**  **^13^C fraction** |
| --- | --- | --- | --- | --- |
| **Gln** | Positive | [M+H]^+^ : 147 | 130 | M0 : 147 → 130 |
|  |  |  |  | M+1 : 148 → 131 |
|  |  |  |  | M+2 : 149 → 132 |
|  |  |  |  | M+3 : 150 → 133 |
|  |  |  |  | M+4 : 151 → 134 |
|  |  |  |  | M+5 : 152 → 135 |
| **Glu** | Positive | [M+H]^+^ : 148 | 130 | M0 : 148 → 130 |
|  |  |  |  | M+1 : 149 → 131 |
|  |  |  |  | M+2 : 150 → 132 |
|  |  |  |  | M+3 : 151 → 133 |
|  |  |  |  | M+4 : 152 → 134 |
|  |  |  |  | M+5 : 153 → 135 |
| **αKG** | Negative | [M-H] ^-^ : 145 | 101 | M0 : 145 → 101 |
|  |  |  |  | M+1 : 146 → 101, 102 |
|  |  |  |  | M+2 : 147 → 102, 103 |
|  |  |  |  | M+3 : 148 → 103, 104 |
|  |  |  |  | M+4 : 149 → 104, 105 |
|  |  |  |  | M+5 : 150 → 105 |
| **IMP** | Positive | [M+H]^+^ : 349 | 137 | M0 : 349 → 137 |
| **UMP** | Negative | [M-H] ^-^ : 323 | 211 | M0 : 323 → 211 |
| **Metabolites** | **Scan mode** | **Precursor ions** | **Fragment ions** | **Ion transition of**  **^15^N fraction** |
| **Gln** | Positive | [M+H]^+^ : 147 | 130 | M0 : 147 → 130 |
|  |  |  |  | M+1 : 148 → 131 |
| **Glu** | Positive | [M+H]^+^ : 148 | 130 | M0 : 148 → 130 |
|  |  |  |  | M+1 : 149 → 131 |
| **αKG** | Negative | [M-H] ^-^ : 145 | 101 | M0 : 145 → 101 |
| **IMP** | Positive | [M+H]^+^ : 349 | 137 | M0 : 349 → 137 |
|  |  |  |  | M+1 : 350 → 138 |
|  |  |  |  | M+2 : 351 → 139 |
|  |  |  |  | M+3 : 352 → 140 |
|  |  |  |  | M+4 : 353 → 141 |
| **UMP** | Negative | [M-H] ^-^ : 323 | 211 | M0 : 323 → 211 |
|  |  |  |  | M+1 : 324 → 211 |
|  |  |  |  | M+2 : 325 → 211 |

**Table S3.** shRNA and sgRNA sequences

| **Name (TRC clone ID)** | **Sequence** | **Validation IB image** |
| --- | --- | --- |
| **shNT** | CCTAAGGTTAAGTCGCCCTCG | Figure 1B |
| **shHDAC6 #1** (TRCN0000004839) | CATCCCATCCTGAATATCCTT |  |
| **shHDAC6 #2** (TRCN0000004842) | CGGTAATGGAACTCAGCACAT |  |
| **shHDAC6 #3** (TRCN0000004843) | CCTCACTGATCAGGCCATATT | Figure S3G |
| **shHDAC6 #4** (TRCN0000004840) | GCCTACGAGTTTAACCCAGAA |  |
| **shHDAC6 #5** (TRCN0000314976) | CATCCCATCCTGAATATCCTT | Figure S3H |
| **shUSP9X #1** (TRCN0000007361) | GAGAGTTTATTCACTGTCTTA | Figure 3I |
| **shUSP9X #2** (TRCN0000007362) | CGATTCTTCAAAGCTGTGAAT |  |
| **shGS** (TRCN0000343990) | AGGAGAAGAAGGGTTACTTTG | Figure 7B |
| **sgNT** | AGCTCGCCATGTCGGTTCTC | Figure 1C |
| **sgHDAC6** | GGTGGAATCCTGGCCGGTTG |  |
| **sgGS #1** | AAATTCCACTCAGGCAACTCTGG | Figure S2D |
| **sgGS #2** | TATTACTGTGGTGTGGGAGC |  |
| **sgUSP15** | TGGTGATGCCCAGTCACTTA | Figure S4G |
